# Potential role of the *Trpv4 c.1491+1G>A* mutation in pulmonary fibrosis in a gene-edited mouse model

**DOI:** 10.3389/fgene.2026.1834091

**Published:** 2026-06-18

**Authors:** Haolong Ruan, Haobo Wang, Cheng Zhu, Zhihao Li, Yuebumu ASu, Zihan Ji, Yong Li, Lantao Gu, Can Fu, Honghao Yu, Pengpeng Yue

**Affiliations:** 1 Engineering Research Center of Rare Disease Prevention and Control, University of Guangxi, Guilin Medical University, Guilin, China; 2 Guangxi Key Laboratory of Drug Discovery and Optimization, Guilin Medical University, Guilin, China; 3 School of Intelligent Medicine and Biotechnology, Guilin Medical University, Guilin, China; 4 Information Center of Guilin Medical University, Guilin, China

**Keywords:** gene editing, lung fibrosis, mouse, single cell transcriptomics, TRPV4

## Abstract

**Introduction:**

TRPV4 is a non-selective cation channel of the TRPV family and plays a key role in fibrosis, but its pathological mechanisms in genetically susceptible individuals remain unclear. This study aimed to investigate the potential role of the *Trpv4* c.1491+1G>A splice-site mutation in pulmonary fibrosis using a gene-edited mouse model.

**Methods:**

The mutation was identified in a family with autosomal dominant familial digital arthropathy-brachydactyly (FDAB). A corresponding gene-edited mouse model was generated using CRISPR/Cas9 technology. Histopathological analysis, single-cell RNA sequencing (scRNA-seq), qPCR, Western blot, and immunofluorescence co-staining were employed to assess phenotypic, transcriptomic, and molecular changes in the lungs.

**Results:**

The model mice exhibited skeletal abnormalities and multi-organ damage, with pronounced pulmonary fibrosis. In the lung tissues of homozygous mutant (*Trpv4*-Hom) mice, wild-type *Trpv4* expression was significantly reduced, accompanied by thickened alveolar septa, pulmonary congestion, and increased collagen deposition. scRNA-seq revealed a decrease in the proportions of alveolar macrophages and NK cells, while Clara cells, fibroblasts, mesothelial cells, and alveolar type II cells increased. The ALCAM-CD6 and MIF-CD74 signaling axes were significantly upregulated. qPCR confirmed the transcriptional upregulation of *Alcam*, *Cd6*, *Cd74*, and *Mif*; Western blot further validated the increased protein levels of ALCAM, CD6, and CD74; and immunofluorescence confirmed the enhanced co-localization of MIF and CD74 in lung tissue.

**Conclusion:**

The *Trpv4* c.1491+1G>A mutation is associated with exacerbated pulmonary fibrosis, altered lung cellular composition, disrupted ALCAM-CD6 immune regulatory pathways and MIF signaling homeostasis, and enhanced pro-fibrotic cell communication. These findings provide novel molecular targets for the development of anti-fibrotic therapies targeting this pathway.

## 1 Introduction

Lung fibrosis is a complex chronic disease characterized by excessive accumulation of extracellular matrix (ECM) components within tissue organs. Its pathological hallmark is the progressive replacement of normal lung tissue with fibrotic tissue, leading to diminished lung function and progressive respiratory impairment (Kolahian et al., 2016; Ortiz-Zapater et al., 2022; Upagupta et al., 2018). Although studies have demonstrated that the development of lung fibrosis is regulated by various cytokines and signaling pathways, current clinical interventions such as pirfenidone and nintedanib (Chianese et al., 2024; Kou et al., 2024), only alleviate symptoms and slow disease progression; thus, achieving a complete cure remains a significant challenge.

The *TRPV4* (Transient Receptor Potential Vanilloid 4) gene is located on human chromosome 12q23–q24.1 and encodes a calcium ion channel protein composed of 871 amino acids. TRPV4 is widely expressed in various tissues including bone (Naert et al., 2019), lung (S et al., 2016), heart (Adapala et al., 2023), blood vessels (Senadheera et al., 2013), kidneys (Kassmann et al., 2012), and skin (Dutta, 2019), and is involved in numerous physiological processes such as fluid osmotic regulation (Endesh et al., 2023; Liedtke, 2005), lung ciliary motility (Sanchez et al., 2017), bone development and remodeling (Nishimura et al., 2012), vascular tone regulation (Hamanaka et al., 2007; Mendoza et al., 2009), temperature and pain sensation, neuronal excitability, and oxidative stress-induced cell death (Bai and Lipski, 2010; Zhong et al., 2023). Recent studies have demonstrated that TRPV4 plays a crucial regulatory role in fibrotic diseases including lung, hepatic, myocardial, and renal fibrosis (Fu et al., 2019; Okada et al., 2022). TRPV4 modulates cellular activity, proliferation, migration, differentiation, and apoptosis by regulating calcium ion influx. Aberrant calcium influx can lead to fibroblast proliferation, thereby promoting fibrosis progression. The disease-relevant human evidence has shown that TRPV4 channel activity is increased in lung fibroblasts derived from patients with idiopathic lung fibrosis (IPF), and that genetic or pharmacological inhibition of TRPV4 attenuates stiffness-dependent myofibroblast differentiation and experimental lung fibrosis, supporting TRPV4 as a clinically relevant profibrotic mechanosensor (Rahaman et al., 2014).

Although TRPV4, as a mechanosensitive calcium channel, has been reported to participate in the fibrosis of extrapulmonary tissues, such as the liver and kidney (Darby et al., 2016; Rosenbaum et al., 2020; Zhang et al., 2025), its molecular network mechanisms mediating lung fibrosis remain to be elucidated. We first identified a pathogenic *TRPV4 c.1491+1G>A* splice-site mutation in an autosomal dominant hereditary disease pedigree. Using CRISPR/Cas9, this mutation was introduced into the homologous mouse locus to generate the *Trpv4*-Hom mouse model, which exhibits skeletal abnormalities and multi-organ pathological damage including lung, kidney, and liver, with prominent lung fibrosis. To further investigate the potential mechanisms associated with the Trpv4 c.1491 + 1G>A mutation and lung fibrosis, we conducted a mechanistic investigation by integrating single-cell RNA sequencing (scRNA-seq).

The breakthrough advancement of single-cell RNA sequencing (scRNA-seq) technology provides a powerful tool to unravel cellular heterogeneity and mechanisms in complex diseases such as lung fibrosis. Peyser et al. (2019) identified profibrotic activated fibroblast subpopulations via scRNA-seq, while Habermann et al. (2020) revealed the critical role of alveolar epithelial cell transdifferentiation toward a profibrotic phenotype. Multiple studies have validated the indispensable role of this technology in deciphering cellular mechanisms of lung fibrosis. Compared with conventional bulk RNA sequencing, scRNA-seq enables precise mapping of gene expression profiles at single-cell resolution, uncovering hidden cell subpopulations and dynamic state transitions (Qin et al., 2023; Qu et al., 2023; Xie et al., 2024). Based on this, the present study integrates histopathological analysis and scRNA-seq technology to comprehensively characterize cellular heterogeneity, differential gene expression, perturbed functional pathways, and cell-cell communication in the lungs of *Trpv4 c.1491+1G>A* mice. Key differential molecules were identified and further validated at the transcriptional level by qPCR and protein level by Western blot, with their expression and colocalization in lung tissue confirmed by co-immunofluorescence staining. The findings deepen our understanding of TRPV4’s pathological functions and may provide molecular targets for antifibrotic therapies targeting specific cell populations and signaling pathways.

## 2 Methods

### 2.1 Animals

All C57BL/6J mice used in this study (male, 8–10 weeks old; n = 3 per group, total n = 6) were specific pathogen-free (SPF) grade and purchased from Hunan SJA Laboratory Animal Co., Ltd. Animals were housed at the Animal Experiment Center of the School of Intelligent Medicine and Biotechnology, Guilin Medical University, under standard SPF conditions. Environmental parameters were maintained at a constant temperature (23 °C–24 °C), relative humidity of 40%–60%, and a 12-h light/dark cycle (lights on: 8:00–20:00; lights off: 20:00–8:00). Mice had *ad libitum* access to sterile water and standard rodent chow.

All experimental procedures were approved by the Animal Ethics Committee of Guilin Medical University (Approval No. GLMC202103279) and conducted in accordance with the ARRIVE guidelines. Animal handling was performed with efforts to minimize stress and discomfort, and all terminal procedures were carried out via humane euthanasia by cervical dislocation.

### 2.2 *TRPV4* mutation analysis of family pedigree

**FIGURE 1:**
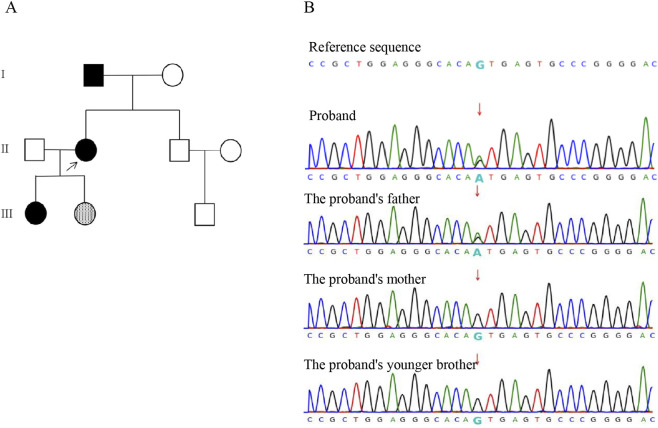
Overview of the FDAB family. **(A)** Pedigree of the FDAB family. Filled symbols represent confirmed patients, the gray-shaded symbol indicates a suspected patient, and the arrow denotes the proband. **(B)** Sanger sequencing chromatograms showing the variant site in the FDAB family.

A three-generation family with a hereditary disorder was identified in this study (Figure 1A), exhibiting clinical features consistent with familial brachydactyly. The pedigree includes the proband, her father, mother, brother, elder daughter, and younger daughter, comprising nine individuals. The proband, a 34-year-old woman, presented with atrophic joint deformities in the left index finger, right thumb, and little finger. Her elder daughter experienced distal atrophy of the left and right ring fingers at birth, which autoamputated within 3 days, making her the youngest affected individual. Other phenotypic features, including facial appearance, intelligence, height, weight, and skeletal structure, were normal among all family members. The inheritance pattern was consistent with autosomal dominant transmission.

To identify the causative mutation within the family, peripheral blood samples were collected from the proband, her father, mother, younger brother, and elder daughter after obtaining informed consent. The samples were then submitted to BerryGenomics Co., Ltd. for genetic variation analysis (Figure 1B).

*TRPV4* mutation analysis was approved by the Medical Ethics Committee of Guilin Medical University (Approval Code: GLLL2021030).

### 2.3 Design of sgRNA and ssODN targeting *Trpv4 c.1491+1G>A*

In this study, a novel pathogenic splice-site mutation, *TRPV4* c.1491 + 1G>A, was identified in a family with an autosomal dominant familial digital arthropathy-brachydactyly (FDAB). To investigate the pathogenic mechanism of this mutation, we employed CRISPR/Cas9-mediated precise genome editing to introduce the human-equivalent variant into the homologous locus of the mouse *Trpv4* gene. Sequence alignment confirms that this position is evolutionarily conserved and functionally equivalent between the human and mouse genomes. This strategy successfully generated gene edited mouse models carrying the *Trpv4* c.1491 + 1G>A mutation, which included both homozygous and heterozygous genotypes.

Sequence analysis of the mouse *Trpv4* gene (ENSMUST0000071968.9) revealed that the c.1491+1G position corresponds to the first nucleotide of intron 8 and lies within a conserved splice donor site. We designed a single-guide RNA (sgRNA) targeting this region and synthesized a single-stranded oligodeoxynucleotide (ssODN) carrying the desired mutation to serve as a homology-directed repair template. The ssODN introduced the G>A substitution at position c.1491 + 1 and an additional synonymous mutation at c.1282 in exon 8 (p.Leu428Leu, G>C) to disrupt the protospacer adjacent motif (PAM) sequence required for CRISPR/Cas9 recognition, thereby preventing re-cutting after repair.

**FIGURE 2:**
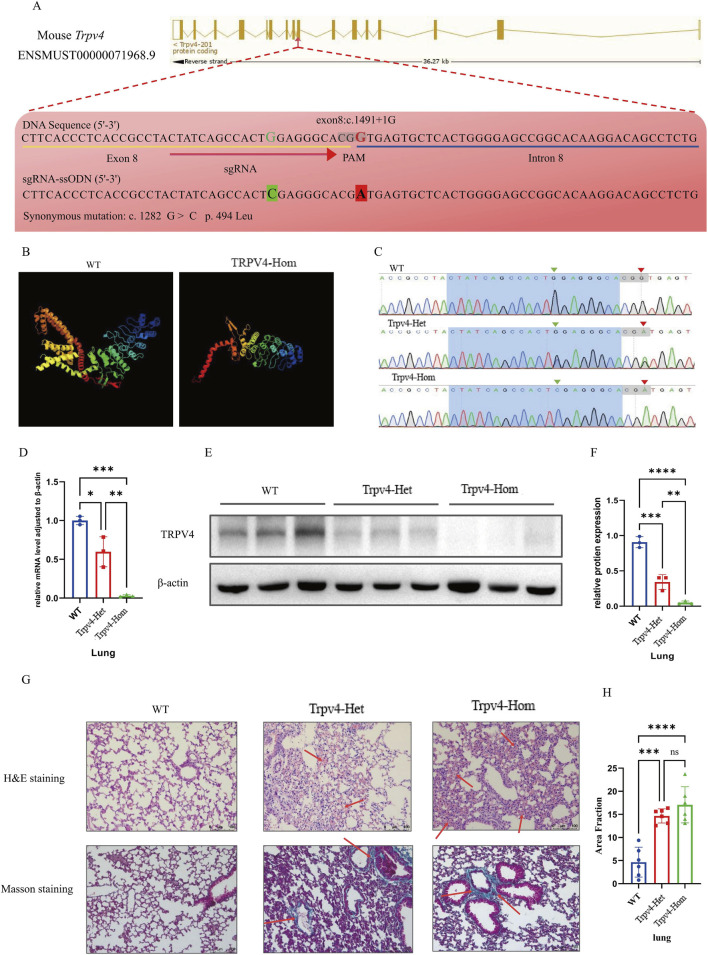
Construction of *Trpv4* gene edited mouse model and phenotypic analysis. **(A)** Schematic diagram of *Trpv4* c.1491 + 1G>A gene editing. **(B)** Three-dimensional structure of the TRPV4 protein. **(C)** DNA sequencing chromatogram. The blue background indicates the Trpv4 sgRNA sequence, and the red label marks the gene editing site. **(D)** Relative mRNA expression of *Trpv4* in the lung (n = 3). **(E)** Western blotting analysis of TRPV4 protein expression (n = 3). **(F)** Immunodetection of TRPV4 protein expression in lung tissue (n = 3). **(G)** H&E and Masson staining of lung tissue sections from mice of the three genotypes **(H)** Quantitative comparison of fibrotic areas in lung tissue among the three genotypes of mice.

The c.1491 + 1G>A mutation disrupts the canonical splice donor site, resulting in exon mis-splicing and premature termination of the open reading frame, producing a truncated mutant protein of 721 amino acids residues. To assess structural consequences, we used the Phyre2 protein structure prediction tool to model and compare the three-dimensional conformations of wild-type and mutant TRPV4 proteins (Figure 2).

### 2.4 Construction and genotypic validation of *Trpv4 c.1491+1G>A* gene edited mouse via CRISPR-Cas9

Cas9 protein (A50575) was purchased from Thermo Fisher, and both sgRNA and ssODN were chemically synthesized. Solutions containing sgRNA (200 ng/μl), ssODN (100 ng/μl), and Cas9 protein (200 ng/μl) were prepared and microinjected into fertilized mouse zygotes. Fertilized embryos were obtained via superovulation and *in vitro* fertilization. Using a micromanipulation system, the mixture of sgRNA, ssODN, and Cas9 protein was injected into the cytoplasm of pronuclear-stage embryos. The injected embryos were cultured *in vitro* to the two-cell stage and then transferred into the oviducts of pseudopregnant female mice to generate embryo transfer-derived mice.

To determine the *Trpv4* genotypes of the resulting mice, tail tissues were collected and digested overnight in lysis buffer containing 0.4 M NaCl, 2 µM EDTA, 1% SDS, 10 µM Tris-HCl, and 100 µg/ml proteinase K. Genomic DNA was extracted from the lysates using phenol-chloroform and recovered by ethanol precipitation. The isolated genomic DNA was used as a template for PCR amplification of the target region using specific primers (Supplementary Table S1). PCR products were purified using a DNA purification kit (AP-PCR-50, Axygen, NY, United States) and subjected to Sanger sequencing. Following genotyping and breeding, homozygous *Trpv4* c.1491 + 1G>A mice were referred to as *Trpv4*-Hom, heterozygous mice as *Trpv4*-Het, and wild-type littermates as WT.

### 2.5 Analysis of wild-type *Trpv4* gene expression levels

Real-time quantitative PCR (qPCR) and Western blotting (WB) were used to evaluate *Trpv4* mRNA and protein expression levels in the lung tissues of *Trpv4*-Het and *Trpv4*-Hom mice.

Total RNA was extracted from lung tissues of *Trpv4*-Het,*Trpv4*-Hom and wild-type (WT) mice for qPCR analysis. Using 1 μg of RNA as a template, cDNA was synthesized with a reverse transcription kit (RR047A, TaKaRa). qPCR reactions were prepared according to the TB Green® Ex Taq™ II kit instructions (RR820L, TaKaRa). The thermal cycling conditions were: initial denaturation at 95 °C for 3 min, followed by 40 cycles of 95 °C for 10 s and 55 °C for 30 s. All expression levels were normalized to β-actin, and relative expression was calculated using the 2^−ΔΔCT^ method. Primer sequences are listed in Supplementary Table S2.

Western blotting was performed to evaluate TRPV4 protein expression in lung tissues of *Trpv4*-Het,*Trpv4*-Hom and WT mice. Tissues were lysed on ice using RIPA buffer (Bestbio, China) supplemented with protease inhibitors. Lysates were centrifuged at 13,800 × g for 15 min at 4 °C, and the supernatants were collected. Equal amounts of protein (60 μg) were separated by SDS-PAGE and transferred onto PVDF membranes. Membranes were incubated overnight at 4 °C with primary antibodies against TRPV4 (ab39260, Abcam, Cambridge, United Kingdom; 1:2000) and β-actin (10494-1-AP, Proteintech; 1:5000). After washing, membranes were incubated with HRP-conjugated goat anti-rabbit secondary antibodies (R&D Systems, United States), developed using an enhanced chemiluminescence kit (EasySee Western Blot Kit, China), and visualized with an imaging system (Bio-Rad, Universal Hood II, United States).

### 2.6 Hematoxylin and eosin (H&E) and masson staining

Lung tissues from WT, *Trpv4*-Het, and *Trpv4*-Hom mice were fixed in 4% paraformaldehyde for 48 h and rinsed with running water for 30 min. Tissues were dehydrated through a graded ethanol series (75% and 85% for 1 h each; 95% and 100% for 2 h each) and cleared twice in xylene for 30 min. Samples were embedded in paraffin after sequential immersion in soft wax (50 °C–52 °C for 1 h) and hard wax (58 °C–60 °C, 2 × 1 h). Longitudinal sections were cut at a thickness of 4 μm, mounted on slides, and baked at 65 °C for 3–4 h. H&E Staining: Sections were deparaffinized in xylene (2 × 5 min) and rehydrated through graded alcohols (100%–75%). Slides were stained with Harris hematoxylin for 2–5 min, differentiated for 10–60 s, and counterstained with eosin for 30 s to 2 min. After rapid dehydration and clearing in xylene, sections were mounted with neutral resin. Masson’s Staining: Following deparaffinization, sections were stained with Weigert’s iron hematoxylin for 5–10 min and differentiated with acid ethanol. After blueing in Masson’s blue solution, sections were treated with Ponceau-fuchsin for 5–10 min and phosphomolybdic acid for 1–2 min. After rapid dehydration and clearing in xylene, sections were mounted with neutral resin. Finally, sections were stained with aniline blue for 1–2 min to visualize collagen fibers in blue. All histopathological slides were examined under a light microscope. Representative images were captured at ×20 (total magnification ×200) for a panoramic view of lung injury.

### 2.7 Preparation of single-cell samples and construction of RNA sequencing libraries

Lung tissue samples were collected from two homozygous *Trpv4*-Hom group and two WT mice. After rinsing in precooled RPMI 1640 medium, tissues were dissociated into single-cell suspensions using SeekGene Tissue Dissociation Reagent A. Red blood cells were removed (Solarbio R1010), and cell viability and debris levels were assessed to determine whether dead cell and debris removal was necessary (Miltenyi 130-109-398/130-090-101). Fresh cells were washed twice in RPMI 1640 and resuspended at a concentration of 1 × 10^6 cells/mL in 1× PBS containing 0.04% bovine serum albumin.

Single-cell RNA sequencing (scRNA-seq) libraries were prepared using the SeekOne® Digital Droplet Single Cell 3′Library Preparation Kit. Cells were mixed with reverse transcription reagents and loaded together with barcoded hydrogel beads (BHBs) and partitioning oil into SeekOne® chip wells. Emulsion droplets were generated, followed by reverse transcription at 42 °C for 90 min and inactivation at 80 °C for 15 min. The resulting cDNA was purified, PCR-amplified, cleaned, fragmented, end-repaired, A-tailed, and ligated with sequencing adapters. Index PCR was used to amplify the 3′polyA-containing DNA fragments harboring cell barcodes and unique molecular identifiers. The indexed libraries were purified and quantified (KAPA Biosystems KK4824) before sequencing on the Illumina NovaSeq 6000 platform with PE150 paired-end reads.

### 2.8 Processing of raw data

Raw single-cell sequencing data were processed using an internal pipeline to generate gene expression profiles. Initially, the data were converted into FASTQ format and subjected to quality control using fastp, which involved removal of low-quality reads, adapter sequences, and poly-A tails. Clean reads were then further filtered and quantified using the SeekSoul® platform. Reads were aligned to the reference genome GRCm38 (mm10) using STAR, and those sharing the same cell barcode, UMI, and gene identity were aggregated to compute UMI counts per gene per cell. The resulting UMI count matrix was used for downstream analysis including cell type identification and clustering using the Seurat package.

### 2.9 Validation of single-cell RNA-seq findings

To validate key targets identified by scRNA-seq, the mRNA and protein expression levels of ALCAM, CD6, CD74, and MIF were examined in lung tissues of Trpv4-Hom and WT mice using qPCR and Western blot (WB), respectively. The spatial colocalization of ALCAM–CD6 and MIF–CD74 was assessed by immunofluorescence (IF) analysis. qPCR primers are listed in Supplementary Table S3; the WB procedure follows the protocol described in Section 2.5. For IF, lung tissues were fixed in 4% paraformaldehyde, paraffin-embedded, and sectioned at a thickness of 4 μm. Antigen retrieval was performed using sodium citrate buffer at 95 °C for 15 min, followed by blocking with 5% BSA at room temperature for 45 min to reduce nonspecific binding. Sections were then incubated overnight at 4 °C with primary antibodies (Servicebio, 1:200). On the following day, fluorescent secondary antibodies (1:500) were applied for 1 h at room temperature. DAPI was used to stain nuclei, and imaging was performed using a confocal microscope.

### 2.10 Downstream data analysis

The scRNA-seq data were analyzed for cell identification and clustering using the Seurat package (Butler et al., 2018; Stuart et al., 2019). First, the UMI count matrix was loaded into an R session using the function Read10X, constructing Seurat objects for both *Trpv4*-Hom and WT samples. Cells with UMI counts less than 30,000, gene counts between 200 and 5,000, and mitochondrial content less than 10% were filtered. Gene expression data were log-normalized and scaled using default parameters. The top 2000 most variable genes were identified using the Seurat function “FindVariableFeatures” for principal component analysis (PCA). The data were scaled using “ScaleData,” and clustering analysis was performed based on the top 20 principal components (PCs) selected via ElbowPlot. The “FindNeighbors” and “FindClusters” functions in Seurat were used to identify cell clusters, with default parameters and a resolution parameter set to 0.5. The clustering results were visualized using Uniform Manifold Approximation and Projection (UMAP). Cell types for each cluster were determined by comparing differentially expressed genes identified by the FindAllMarkers function with known cell marker genes from the literature (Guo et al., 2023; Negretti et al., 2021), as well as the CellMarker database and PanglaoDB database The FindMarkers function was used to identify differentially expressed genes between *Trpv4*-Hom and WT groups within each cell type, with the parameter min. pct set to 0.25. Gene Ontology (GO) enrichment analysis of differentially expressed genes was performed using the R package clusterProfiler (v 3.12.0), and the results were visualized with Dotplot, showing enriched biological pathways in different cell types. Cell communication analysis was conducted using CellChat (v 1.1.3), with the raw expression matrices of *Trpv4*-Hom and WT groups as inputs (Jin et al., 2021).

### 2.11 Quantification and statistical analysis

All experimental data are presented as mean ± standard deviation (SD). Statistical analyses were performed using GraphPad Prism 10 or R software. Comparison of multiple groups: For comparisons involving more than two groups, one-way analysis of variance (ANOVA) followed by Tukey’s post-hoc test was used to determine statistical significance. Comparison of two groups: For comparisons between two independent groups, a two-tailed Student’s t-test was employed. Significance levels: The following thresholds were used to denote statistical significance: *p < 0.05, **p < 0.01, ***p < 0.001, and ****p < 0.0001.

## 3 Results

### 3.1 Discovery of a novel pathogenic *TRPV4* c.1491 + 1G>A splicing-site mutation in a clinical pedigree

We identified a three-generation family with the disease (Figure 1A), whose clinical manifestations of progressive joint atrophy and loss were consistent with those of FDAB. Bioinformatics analysis of the proband’s whole exome sequencing (WES) data, combined with family clinical assessment and database query (Human Gene Mutation Database and ClinVar), revealed a novel splicing site variation (c.1491 + 1G>A) on exon 8 of *TRPV4*. This variation was subsequently verified by Sanger sequencing (Figure 1B). The proband inherited the mutation from her father, yet her mother and younger brother did not carry this specific variant.

### 3.2 Construction and phenotypic analysis of *Trpv4 c.1491+1G>A* gene edited mouse

A *Trpv4* gene edited mouse model carrying the c.1491 + 1G>A splicing-site mutation was generated via CRISPR/Cas9-mediated genome editing, involving zygote microinjection and embryo transfer. Successful introduction of the mutation was confirmed by Sanger sequencing at the target locus (Figure 2A). Through heterozygous breeding, both *Trpv4*-Het heterozygous and *Trpv4*-Hom homozygous mice were obtained. Bioinformatic analysis revealed a truncated protein sequence (Supplementary Table S3) and substantial changes in protein structure (Figures 2B,C) due to a coding-region disruption in the *Trpv4*-Hom allele. Compared with WT mice, *Trpv4*-Hom mice exhibited markedly reduced expression of wild-type *Trpv4* mRNA and protein (Figures 2D–F). Histological examination by H&E staining showed extensive red blood cell infiltration in alveoli and interstitium, thickened alveolar septa, and alveolar wall rupture or fusion. Masson’s trichrome staining indicated pronounced lung fibrosis in *Trpv4*-Hom group (Figures 2G,H). The red arrows in Figure 2G indicate the sites of red blood cell infiltration and collagen deposition associated with pulmonary fibrosis. Raw data are available in Supplementary Material R (WB + qPCR + H&E + Masson + R.zip).

These results indicate the successful establishment of a clinically relevant mouse model exhibiting a phenotype consistent with spontaneous pulmonary fibrosis and skeletal abnormalities.

### 3.3 Identification and differentiation of cell types in lung tissue of *Trpv4* c.1491 + 1G>A gene edited mouse

**FIGURE 3:**
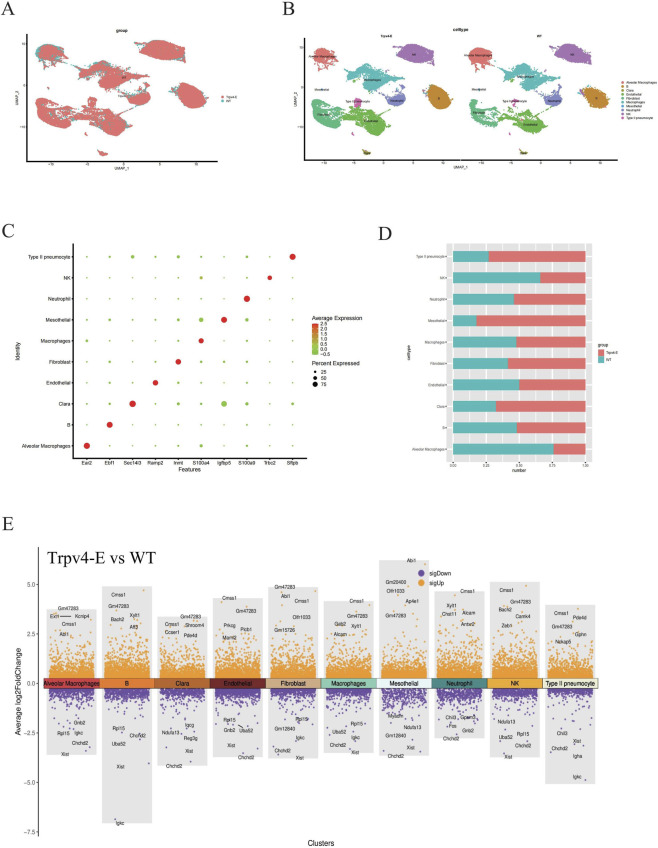
Differential genes and cell proportion in mouse lung tissue. **(A)** UMAP scatter plots visualizing the *Trpv4*-Hom and WT groups, with cells from the same sample marked in the same color. **(B)** UMAP visualization of scRNA-seq data from *Trpv4*-Hom (n = 25,426) and WT (n = 29,332) mouse lungtissues. UMAP projections of *Trpv4*-Hom mouse lung tissue samples (left)and WT mouse lung tissuesamples (right). **(C)** Dot plot showing the expression of known gene markers in different cell types. **(D)** Proportions of each cell type in the *Trpv4*-Hom and WT groups. **(E)** Volcano plot displaying significant differences in gene expression between *Trpv4*-Hom and WT samples.

Following quality control and batch effect correction (Figure 3A), a total of 25,426 cells were obtained from *Trpv4*-Hom homozygous mouse lung tissues and 29,332 cells from WT lung tissues, representing 23,700 mouse genes for downstream analysis (Supplementary Figures S1–S3). UMAP visualization identified 22 transcriptionally distinct clusters (Supplementary Figures S4–S5). Based on the expression of well-established marker genes in murine lungs, 10 major cell types were annotated: alveolar macrophages (Ear2), B cells (Ebf1), Clara cells (Sec14l3), endothelial cells (Ramp2), fibroblasts (Inmt), macrophages (S100a4), mesothelial cells (Igfbp5), neutrophils (S100a9), NK cells (Trbc2), and alveolar type II (AT2) cells (Sftpb) (Figures 3B,C; Supplementary Table S4). The cell-type compositions of *Trpv4*-Hom and WT lungs were compared to assess the impact of the c.1491 + 1G>A mutation. All 10 cell types were present in both groups. However, *Trpv4*-Hom lungs exhibited a marked reduction in the proportion of alveolar macrophages and NK cells, along with a significant increase in Clara cells, fibroblasts, endothelial cells, and AT2 cells. Proportions of B cells, endothelial cells, macrophages, and neutrophils remained relatively unchanged (Figure 3D; Supplementary Figure S6). Transcriptomic analysis identified the top five upregulated and top five downregulated genes within distinct cell types between groups (Figure 3E). Upregulated genes were mainly associated with cell adhesion, migration, signal transduction, metabolism, and stress response—processes critical for lung injury repair and fibrogenesis. Conversely, downregulated genes were enriched in pathways related to protein synthesis, mitochondrial function, immune regulation, and cell survival, suggesting that their impairment may exacerbate lung injury and fibrosis. These findings suggest that *Trpv4* gene editing is associated with alterations in immune cell homeostasis and alters metabolic function. The *Trpv4* mutation is associated with a remodeling of the lung cellular landscape, suggesting a shift toward a profibrotic cell composition.

### 3.4 Enrichment analysis of differentially expressed genes

**FIGURE 4:**
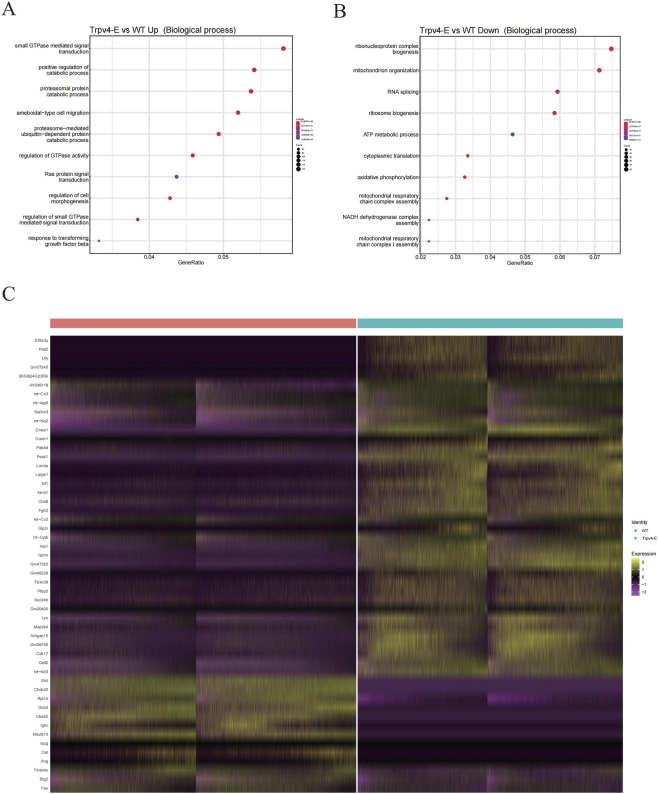
Enrichment analysis of differentially expressed genes in mouse lung tissue. **(A)** GO enrichment analysis of upregulated genes in *Trpv4*-Hom versus WT lung tissue. **(B)** GO enrichment analysis of downregulated genes in *Trpv4*-Hom versus WT lung tissue. **(C)** Heatmap of differentially expressed genes between *Trpv4*-Hom and WT lung tissues.

Differential gene expression analysis between the *Trpv4*-Hom and WT groups revealed that genes upregulated in the *Trpv4*-Hom lungs were predominantly enriched in biological processes related to cell migration, inflammatory responses, protein homeostasis, and signal transduction (Figure 4A). These pathways may contribute to tissue repair following lung injury, but could also exacerbate fibrosis by activating pro-fibrotic signals such as TGF-β. Conversely, genes downregulated in the *Trpv4*-Hom group were mainly associated with energy metabolism, protein synthesis, and mitochondrial function (Figure 4B). Impairment of these processes typically compromises cellular repair mechanisms and functional integrity, potentially aggravating lung injury. Collectively, the transcriptional alterations observed (Figure 4C) suggest that regulation of these pathways plays a crucial role in maintaining cellular metabolism, signaling, structural integrity, and repair capacity. These findings imply that *Trpv4* gene editing may predispose lung tissue to injury and fibrotic remodeling. These transcriptomic alterations suggest that the *Trpv4* mutation triggers a transition from normal metabolic homeostasis to inflammatory and migratory cellular programs.

### 3.5 CellChat analysis reveals different cell interaction patterns

**FIGURE 5:**
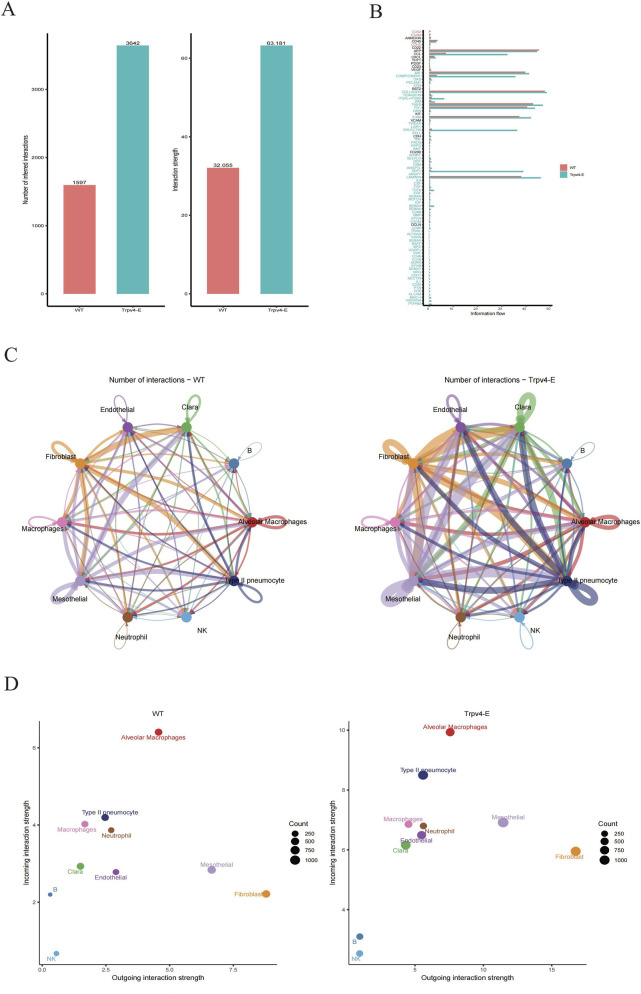
Overall differences in cell–cell communication in mouse lung tissue. **(A)** Comparison of the total number and strength of cell–cell communications between WT and *Trpv4*-Hom groups. **(B)** Bar plot showing enriched signaling pathways differing between WT and *Trpv4*-Hom groups. **(C)** Comparison of cell–cell communication events, including intercellular signaling patterns, between WT and *Trpv4*-Hom groups. **(D)** Two-dimensional visualization of outgoing and incoming interaction strengths between WT and *Trpv4*-Hom groups.

By comparing detailed intercellular signaling profiles between groups, we observed a global increase in both the number and strength of interactions in the *Trpv4*-Hom group (Figure 5A). Further pathway enrichment analysis revealed significant differences in signaling pathways between *Trpv4*-Hom and WT lungs (Figure 5B). In particular, several pathways were markedly altered in *Trpv4*-Hom group, including those associated with extracellular matrix organization—such as laminin, collagen, thrombospondin (THBS), and fibronectin (FN1)—as well as inflammation- and fibrosis-related pathways, including SPP1, ICAM, TGF-β, complement, and galectin signaling. To elucidate potential intercellular signaling patterns, we applied the CellChat algorithm to construct statistical and biological communication networks based on key ligand–receptor interactions (Figure 5C; Supplementary Figure S7), which uncovered pronounced differences in cell–cell communication between the WT and *Trpv4*-Hom groups. Notably, the overall interaction strength among all cell types was significantly elevated in the *Trpv4*-Hom lungs compared to WT controls (Figure 5D). Collectively, CellChat analysis reveals that the *Trpv4* mutation amplifies intercellular communication, particularly within pathways associated with extracellular matrix remodeling and inflammatory responses.

### 3.6 Remodeling of B Cell–NK cell communication networks in *Trpv4*-Hom group

**FIGURE 6:**
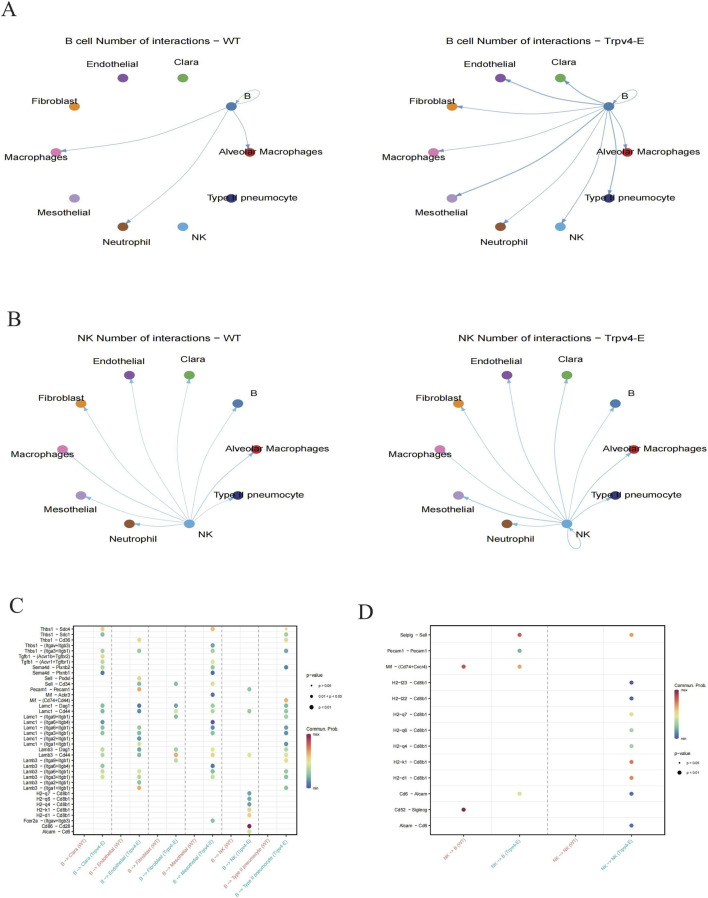
Alterations in intercellular networks and ligand–receptor interactions involving B cells and NK cells. **(A)** Cellular interaction networks between B cells and other cell types in WT and *Trpv4*-Hom groups **(B)** Cellular interaction networks between NK cells and other cell types in WT and *Trpv4*-Hom groups. **(C)** Differential ligand–receptor interactions involving B cells between WT and *Trpv4*-Hom groups. **(D)** Differential ligand–receptor interactions involving NK cells between WT and *Trpv4*-Hom groups.

CellChat analysis revealed significant alterations in communication strength between B cells and several cell types—including Clara cells, endothelial cells, fibroblasts, mesothelial cells, NK cells, and alveolar type II (AT2) epithelial cells—in the *Trpv4*-Hom group compared to WT controls (Figure 6A). Simultaneously, the intercellular signaling network of NK cells also exhibited marked differences between groups (Figure 6B). Further investigation of ligand–receptor interactions identified several signaling pathways significantly altered in B cells, including ALCAM, CD6, CD86, SEMA4, SELL, THBS, TGF-β, Laminin, PECAM1, and MIF pathways (Figure 6C; Supplementary Figure S7). These pathways are potentially involved in modulating B cell activation, adhesion, migration, immune regulation, and extracellular matrix remodeling, thereby contributing to lung inflammation and fibrosis. Correspondingly, changes in NK cell signaling were primarily enriched in ALCAM, CD6, and SELPLG pathways (Figure 6D; Supplementary Figure S7), suggesting that these interactions may regulate NK cell–mediated immune surveillance, cytotoxic responses, and inflammatory modulation, playing a critical role in lung injury and fibrotic progression. These findings indicate that the *Trpv4* mutation reshapes the immune communication network, identifying the ALCAM-CD6 and MIF signaling axes as primary perturbed pathways.

### 3.7 Functional analysis of B and NK cells

**FIGURE 7:**
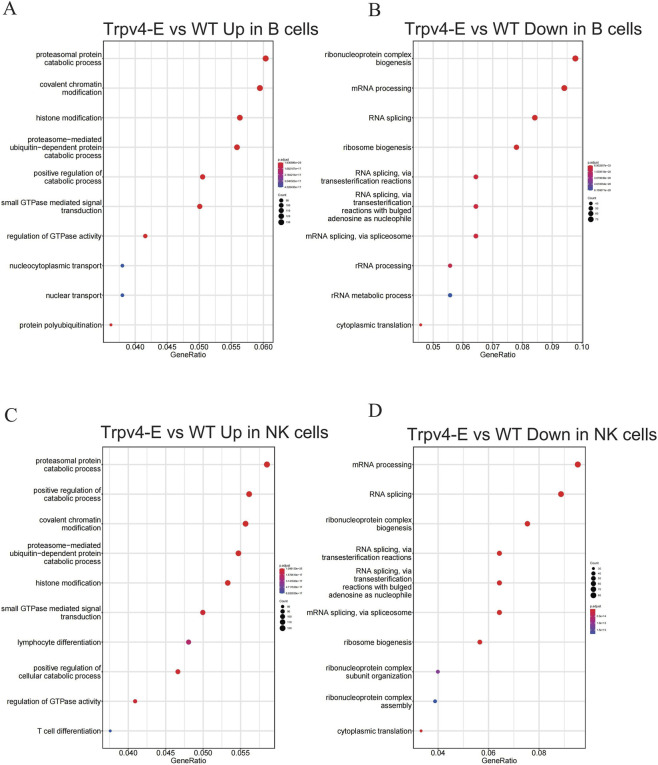
GO enrichment analysis of differentially expressed genes in B cells and NK cells from *Trpv4*-Hom group. **(A)** GO molecular function enrichment analysis of upregulated genes (left) in B cells. **(B)** GO molecular function enrichment analysis of downregulated genes (right) in B cells. **(C)** GO molecular function enrichment analysis of upregulated genes (left) in NK cells. **(D)** GO molecular function enrichment analysis of downregulated genes (right) in NK cells.

In *Trpv4*-Hom group, CellChat analysis revealed markedly altered intercellular communication patterns of B and NK cells compared to the WT group, particularly a significant reduction in NK cell abundance and proportion. To further investigate potential functional changes, we performed functional enrichment analysis of differentially expressed genes (DEGs) in both cell types. In the *Trpv4*-Hom group, upregulated genes in B cells were enriched in biological processes related to chromatin remodeling, including proteasome-mediated degradation, protein ubiquitination, and histone modification, suggesting a possible link to inflammatory responses (Figure 7A). Additionally, activation of small GTPase–mediated signaling pathways indicated enhanced roles in B cell migration and immune modulation. Conversely, downregulated pathways in B cells were associated with mRNA processing, RNA splicing, and ribosome biogenesis, potentially impairing protein synthesis capacity and thus compromising cellular repair and immune function (Figure 7B). Similarly, NK cells in the *Trpv4*-Hom group exhibited activation of pathways involved in proteasomal degradation, chromatin modification, small GTPase signaling, and lymphocyte differentiation, implying a role in immune response regulation (Figure 7C). Meanwhile, NK cells showed suppression of post-transcriptional processes, including mRNA splicing and ribosome formation, which may hinder their effector functionality (Figure 7D).

### 3.8 Validation of single-cell sequencing results by qPCR, Western blot, and immunofluorescence

**FIGURE 8:**
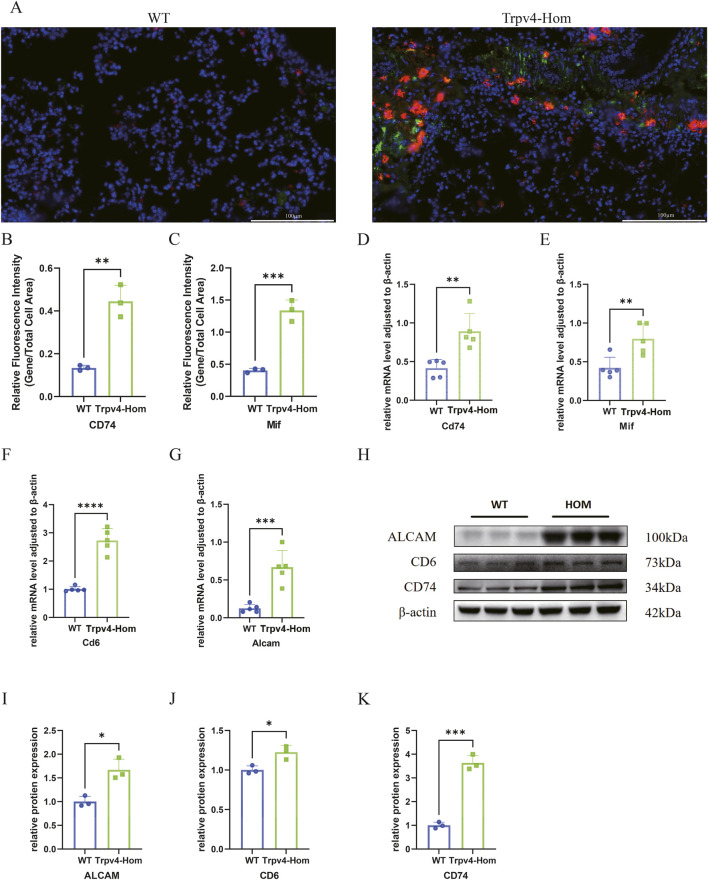
Validation of single-cell RNA-seq findings by qPCR, Western blotting, and immunofluorescence. **(A)** Representative immunofluorescence (IF) images of lung tissues from WT and Trpv4-Hom groups showing the expression of MIF (red) and CD74 (green). Nuclei were counterstained with DAPI (blue). Scale bar = 100 μm. **(B,C)** Quantitative analysis of CD74 **(B)** and MIF **(C)** immunofluorescence signal intensity in each group using ImageJ software (n = 3). **(D–G)** qPCR analysis of *Cd74* **(D)**, *Mif* **(E)**, *Cd6* **(F)**, and *Alcam.* **(G)** mRNA levels in lung tissues of WT and *Trpv4*-Hom groups, normalized to β-actin (n = 5). **(H)** Representative Western blot bands showing ALCAM, CD6, and CD74 protein expression in lung tissues of WT and Trpv4-Hom groups **(I–K)** Statistical summary of the relative protein expression levels of ALCAM **(I)**, CD6 **(J)**, and CD74. **(K)** based on Western blot results (n = 3). Data are presented as mean ± SD. Statistical significance is defined as: *p < 0.05, **p < 0.01, ***p < 0.001, ****p < 0.0001.

Immunofluorescence co-staining revealed that MIF (red) and CD74 (green) were predominantly localized in immune cell-enriched regions of the lung and were markedly intensified in the *Trpv4*-Hom group (Figures 8A–C). qPCR analysis revealed significantly elevated mRNA levels of *Mif*, *Cd74*, *Alcam*, and *Cd6* in the *Trpv4*-Hom group (Figures 8D–G). Western blot further confirmed these changes, showing a marked upregulation of ALCAM and CD6 protein expression in lung tissues from *Trpv4*-Hom group (Figures 8H–K), consistent with the qPCR findings. These results suggest that the *Trpv4* c.1491 + 1G>A mutation may contribute to lung inflammation or structural remodeling by upregulating immune-associated molecules. In conjunction with single-cell transcriptomic data, we observed a pronounced enhancement of two key ligand–receptor signaling axes—ALCAM–CD6 and MIF–CD74—in *Trpv4*-Hom group, particularly within the communication network between B cells and NK cells. The upregulation of these molecules not only reflects enhanced adhesion, activation, and inflammatory signaling between immune cells, but also suggests their critical regulatory roles in the onset and progression of lung fibrosis. In summary, these multi-level validation results confirmed the upregulation of the key components of the MIF-CD74 and ALCAM-CD6 axes, suggesting their potential involvement in the Trpv4-associated fibrotic process.

## 4 Discussion

This study established a *Trpv4* gene edited mouse model based on the splice-site mutation *Trpv4* c.1491 + 1G>A identified in a clinical pedigree, and employed single-cell transcriptomic profiling to investigate the impact of this mutation on various cell types and signaling pathways in lung tissue. Our results revealed that the *Trpv4* c.1491 + 1G>A mutation altered the cellular composition of the lung, characterized by a reduction in immune cells such as macrophages and NK cells, and an increase in structural cell types including fibroblasts, alveolar type 2 (AT2) cells, and mesothelial cells. These changes suggest a strong association with the formation of a profibrotic microenvironment. Moreover, the upregulation of key immune signaling axes such as ALCAM–CD6 and MIF–CD74 suggests that the *Trpv4* mutation may contribute to fibrosis progression and intercellular communication, thereby driving inflammatory responses and alveolar structural damage.

### 4.1 Identification of a clinical mutation and establishment of the *Trpv4* c.1491+1G>A gene edited mouse model

Based on a hereditary family with a well-defined clinical phenotype, this study identified for the first time a novel splice-site mutation (c.1491 + 1G>A) in the *TRPV4* gene. Located at a highly conserved splice donor site, this variant is potentially pathogenic and provides a clinical basis for disease modeling. Whole-exome sequencing of family members confirmed that the mutation is a splice-site variant in exon 8 of *TRPV4 (c.1491+1G>A)*, exhibiting an autosomal dominant inheritance pattern.

Using the CRISPR/Cas9 system, we successfully generated a *Trpv4 c.1491+1G>A* gene edited mouse model. Previous studies on *TRPV4* mutation-related diseases have primarily employed global knockout (Trpv4-KO) or overexpression models (Lan et al., 2021). Although these models have contributed to understanding the physiological functions of TRPV4, their mutation sites do not match those found in clinical settings, making it difficult to precisely dissect the pathogenic mechanisms of patient-specific *TRPV4* mutations. In contrast, the model developed here accurately introduces the clinically identified *TRPV4 c.1491+1G>A* splice-site mutation discovered in a patient family, providing a unique tool for in-depth investigation into the pathogenic mechanisms of *Trpv4* mutations. The *Trpv4* gene edited mouse model established in this study represents the first animal model based on a patient-specific splice-site mutation, holding significant scientific and translational potential.

Bioinformatic analysis suggests that the modeled splice-site mutation can lead to aberrant splicing, resulting in a novel truncated protein with likely altered function. Although the channel kinetics of this protein were not directly examined here, the spontaneous pulmonary fibrosis phenotype observed in the model mice is consistent with the field paradigm that “TRPV4 activity promotes fibrosis” (Darby et al., 2016). We hypothesize that the mutation may be associated with pathogenicity, potentially linked to abnormal activation of the truncated protein—a concept mechanistically aligned with several documented splice-site mutations that result in gain-of-function (GOF) outcomes. These include the *FGFR2-IIc* splice-switching mutation that causes constitutive receptor activation and craniosynostosis; the *GSDME c.1183+1G>A* mutation leading to exon 8 skipping and producing a toxic gain-of-function truncated protein associated with deafness (Rosso et al., 2025); and the *TP53* synonymous mutation that creates a cryptic splice site and generates a proliferation-promoting truncated protein driving tumorigenesis (Austin et al., 2017). These precedents support the possibility that splicing variants can acquire pathogenic activity by producing structurally abnormal, functionally altered proteins, thereby offering a theoretical framework for further elucidating the pathogenic mechanism of the present *TRPV4* mutation.

### 4.2 Impact of the *Trpv4* c.1491 + 1G>A splice-site mutation on AT2 cell proliferation and alveolar injury

In our histological assessment and scRNA-seq profiling, we observed increased representation of AT2 cells in *Trpv4*-Hom lungs together with broader remodeling of the lung microenvironment. This pattern is consistent with an epithelial injury/repair response and suggests that AT2-associated changes may be associated with the fibrotic remodeling observed in this model. In parallel, our single-cell data indicate immune compartment alterations and inflammatory signaling enrichment, which could contribute to epithelial dysfunction and subsequent fibroblast activation during fibrosis progression. Previous studies have implicated AT2 dysfunction/hyperplasia in lung fibrosis and reported that AT2-targeted genetic perturbations can be sufficient to induce spontaneous fibrotic phenotypes in humans and mice (Shao et al., 2024; Ruaro et al., 2021; Parimon et al., 2020; Feng et al., 2023), in line with the epithelial-centered features observed here.

### 4.3 Inflammation and metabolic imbalance mechanisms revealed by differentially expressed genes

Differential gene expression analysis revealed that genes upregulated in the *Trpv4*-Hom group are associated with cell migration, inflammation, protein homeostasis, and signal transduction. These changes may contribute to attenuating lung injury and promoting repair but could also exacerbate fibrosis by activating fibrotic pathways such as TGF-β (Peng et al., 2022; Meng et al., 2016; Hu et al., 2018). Notably, in all cell types from the *Trpv4*-Hom group, Gm47283 and Cmss1 were consistently upregulated. Gm47283 is a non-coding RNA studied for its role in myocardial infarction (MI) (Gao et al., 2022). Cmss1 is an RNA-binding protein potentially involved in tumorigenesis and progression, considered a potential diagnostic and prognostic biomarker for liver hepatocellular carcinoma (LIHC) (Chen et al., 2024). In contrast, genes related to energy metabolism, protein synthesis, and mitochondrial function, such as Xist and CHCHD2, were significantly downregulated in nearly all cell types in the *Trpv4*-Hom group. Xist is a long non-coding RNA responsible for X chromosome inactivation, and its downregulation exacerbates sepsis-induced lung injury (Zhang et al., 2023; Song et al., 2021; Li et al., 2021). Chchd2 is a mitochondrial-associated protein; its reduced expression may impair mitochondrial respiration, affecting cellular energy metabolism (Meng et al., 2017; Gao et al., 2025). These gene expression changes highlight the complexity of cellular adaptation and pathological states, reflecting the potential interplay between repair-related processes and fibrotic changes.

### 4.4 *Trpv4* regulation of intercellular communication and ALCAM/CD6, MIF/CD74 signaling

The number and strength of intercellular interactions were significantly increased in the *Trpv4*-Hom group, especially in pathways related to extracellular matrix (ECM) function and inflammatory responses. These findings suggest that the *Trpv4* c.1491 + 1G>A mutation may enhance intercellular communication and inflammatory responses, which may contribute to the progression of lung fibrosis. Changes in the interactions between B cells and NK cells further support this conclusion, particularly those involving the ALCAM and CD6 signaling pathways.

Activated leukocyte cell adhesion molecule (ALCAM) plays an important role in cell adhesion and signal transduction. Kim et al. found that ALCAM inhibits apoptosis of alveolar epithelial type II cells (AT2 cells) by activating the PI3K-Akt signaling pathway (Kim et al., 2022), thereby exerting a protective effect in the pathogenesis of lung fibrosis. The marked increase in AT2 cell proportion suggests that abnormal activation of ALCAM may lead to an increase in AT2 cell numbers. This phenomenon may be related to cell proliferation and functional changes during fibrosis. In the context of lung fibrosis, although ALCAM protects by inhibiting apoptosis, it may simultaneously promote AT2 cell proliferation or regeneration, resulting in increased cell numbers. Therefore, the increase in AT2 cells may be associated not only with apoptosis inhibition but also with abnormal proliferation, indicating a complex role of ALCAM in lung fibrosis.

CD6 is an immune regulatory molecule involved in interactions between T cells and antigen-presenting cells (Rambaldi et al., 2022; Gurrea-Rubio et al., 2024; Gurrea-Rubio et al., 2025). It is also a potential therapeutic target for multiple sclerosis (MS). Wagner et al. further identified ALCAM and CD6 as risk factors for MS (Wagner et al., 2014). MS is an autoimmune disease that damages the fatty myelin sheath protecting nerve fibers in the brain, leading to neuronal injury and impaired or slowed signal transmission. Thus, *Trpv4* editing may regulate the interactions between B cells and NK cells by affecting intercellular communication and inflammation, particularly via the ALCAM and CD6 signaling pathways, playing a vital role in lung fibrosis. This mechanism resembles alterations in the immune landscape observed in multiple sclerosis.

Moreover, studies have shown that gain-of-function mutations in TRPV4 significantly enhance calcium influx in endothelial cells, severely impairing blood-brain barrier integrity and triggering motor neuron degeneration (Sullivan et al., 2024). This suggests that TRPV4 channel dysfunction directly affects intracellular calcium homeostasis, playing a key role in regulating cell function and inflammatory responses. Conversely, *Trpv4* editing may alter TRPV4 channel expression, affecting cellular responses to mechanical stress and disrupting normal calcium signaling. In lung fibrosis, *Trpv4* editing may importantly influence lung fibroblast function. Fibroblasts are crucial in fibrosis progression. *Trpv4* may affect fibroblast function by modulating normal calcium signaling pathways, leading to increased collagen deposition and exacerbating fibrosis. *Trpv4* editing might also alter immune cell activation states, increasing pro-inflammatory cytokine release and further promoting lung inflammation and fibrosis. This mechanism is analogous to TRPV4-induced blood-brain barrier disruption in endothelial cells, although it occurs in different tissues and disease contexts, both underscoring TRPV4’s key role in regulating cell function and inflammation. *Trpv4* editing may interfere with calcium balance and inflammatory responses via multiple pathways, potentially influencing lung fibrosis.

Unique changes in B cell communication involve CD86, SELL, THBS, PECAM1, TGF-β, MIF, SEMA4, and laminin signaling pathways. Among these, TGF-β is a key regulator of lung fibrosis (Peng et al., 2022; Meng et al., 2016; Hu et al., 2018; Xu et al., 2016), promoting fibrosis formation and progression through extensive studies. Macrophage migration inhibitory factor (MIF) plays a crucial role in inflammatory responses. Yifeng Luo et al. demonstrated that reducing MIF can inhibit lung inflammation and fibrosis in rats (Luo et al., 2021), and in line with this, our study shows elevated MIF levels, suggesting its key role in modulating lung fibrosis. CD86 primarily participates in immune responses and T cell activation. Research indicates that during the inflammatory phase of idiopathic lung fibrosis (IPF), co-stimulatory molecules CD86 and CD80 are upregulated, potentially serving as biomarkers of fibrosis onset. Signaling pathways such as SEMA4 regulate cell migration and tissue remodeling; laminin, a key ECM component, contributes to tissue remodeling and fibrosis. SELL mainly functions in leukocyte adhesion and migration; THBS is involved in ECM remodeling and cell adhesion; PECAM1 operates in vascular endothelial cells, promoting angiogenesis and mediating cell adhesion and signal transduction in inflammatory responses. Although the direct roles of these pathways in lung fibrosis remain unclear, they likely participate in inflammation and fibrosis processes.

NK cells are critical components of innate immunity, primarily responsible for recognizing and killing virus-infected and tumor cells. They regulate immune responses through cytokine and chemokine secretion and interactions with macrophages and dendritic cells. The reduction in the proportion and potential functional impairment of NK cells during lung fibrosis may contribute to an imbalanced immune microenvironment, impaired clearance of damaged cells, dysregulated inflammation, and exacerbated fibrosis. NK cells modulate the immune environment via these signaling pathways, playing vital roles in inhibiting fibrosis and promoting tissue repair. The *Trpv4*-Hom-induced alterations in these pathways may impair NK cell functions, weakening their immune surveillance and inflammation regulation, thus worsening lung fibrosis progression.

The *Trpv4* c.1491 + 1G>A mutation induces lung inflammatory responses and fibrotic features, as demonstrated by B cell and NK cell analyses. We found that signaling pathways in these cells are upregulated, affecting protein synthesis, while the downregulation of protein synthesis pathways significantly slows cell repair, exacerbating inflammation. Enhanced interactions between B cells and NK cells may lead to stronger inflammatory responses. However, despite upregulated signaling pathways, the notable downregulation of protein synthesis suggests impaired self-repair capacity of these cells. Since protein synthesis is critical for cell repair and regeneration, this downregulation may prevent immune cells from effectively repairing damage, sustaining and aggravating inflammation. lung fibrosis is often driven by inflammatory responses, suggesting that *Trpv4*-Hom is associated with immune cell alterations that may favor fibrosis progression.

### 4.5 Validation of single-cell transcriptomic findings

To validate the key signaling pathways suggested by single-cell transcriptomic data, we performed multi-level verification of the MIF-CD74 and ALCAM-CD6 axes at both mRNA and protein levels. Immunofluorescence staining revealed a pronounced co-expression of MIF and its receptor CD74 in immune cell-enriched regions of lung tissue from *Trpv4*-Hom mouse. These findings were further corroborated by qPCR and Western blot analyses, which showed significantly elevated mRNA and protein levels of Mif and Cd74, demonstrating a significant increase in the expression of key components within this signaling axis.

Concurrently, upregulation of the ALCAM–CD6 axis was consistent with the scRNA-seq results, as both transcriptional and protein expressions of Alcam and Cd6 were significantly increased in *Trpv4*-Hom lung tissue. Previous studies have demonstrated that MIF-CD74 signaling facilitates recruitment of macrophages and T cells, while the ALCAM–CD6 axis mediates T cell activation and migration across epithelial barriers, aligning with the observed functional alterations in immune cells in this study.

Notably, single-cell data indicated that ALCAM and CD74 are predominantly enriched in lung epithelial cells—particularly AT2 cells—and stromal immune cells such as macrophages. AT2 cells have been shown to be a key source of epithelial regeneration in lung fibrosis and can upregulate ligands including MIF and ALCAM under stress conditions, thereby promoting immune cell infiltration and amplifying inflammatory responses. Our results strongly support this mechanism, suggesting that the *Trpv4* c.1491 + 1G>A mutation is potentially associated with the remodeling of the lung immune microenvironment and fibrosis progression by disrupting the interplay between AT2 cells and immune cells.

In summary, qPCR, Western blot, and immunofluorescence results consistently corroborate the scRNA-seq findings, confirming the significant upregulation of key molecules in the MIF-CD74 and ALCAM-CD6 signaling axes in *Trpv4*-Hom lung tissue and highlighting their critical roles in mediating immune regulation, inflammation, and tissue remodeling.

### 4.6 Study limitations and future perspectives

While this study has achieved the aforementioned advances, several important limitations remain to be addressed in future work. Firstly, the hypothesis that the *Trpv4 c.1491+1G>A* mutation leads to a truncated protein with “gain-of-function” properties is primarily based on bioinformatic predictions and phenotypic correlations and has not been directly validated at the protein level. Key biophysical and biochemical characteristics of the mutant protein—including its expression, structure, basal channel activity, calcium permeability, and response to ligands or mechanical stimuli—await experimental confirmation. Secondly, the mechanistic insights rely predominantly on animal models, lacking functional validation using patient-derived cells or tissues, which limits the direct interpretation of this mutation’s role in human disease. Furthermore, although the study originated from a clinical family, a systematic assessment of pulmonary fibrosis phenotypes in family members has not been completed. The precise clinical relevance of this mutation to human disease therefore requires further confirmation in larger cohorts.

Looking forward, future research should focus on the following directions: direct characterization of the biochemical and channel functional properties of the mutant TRPV4 protein at the molecular and cellular levels; functional validation at the cellular level; and clarification of the genotype–phenotype association with pulmonary fibrosis in expanded clinical populations. These efforts will contribute to a comprehensive elucidation of the pathogenic mechanism of this novel *TRPV4* splice-site mutation and lay the groundwork for developing targeted therapeutic strategies against *TRPV4*-associated fibrotic diseases.

## 5 Conclusion

This study identified a splice-site mutation in *TRPV4 c.1491+1G>A* in a family with FDAB. Based on this finding, we generated a gene edited *Trpv4* mouse model and performed systematic pathological assessment across major organs. Marked fibrotic remodeling was observed in the lungs. Focusing on lung tissue, single-cell transcriptomic profiling indicated that the *Trpv4* mutation is associated with lung fibrosis, potentially linked to alterations in cellular composition, shifts in immune axes, and enhanced intercellular communication. These findings suggest that the mutation leads to a significant remodeling of the immune landscape, particularly within the B cell and NK cell populations. The upregulated expression of key immune-related molecules (Mif, Cd74, Alcam, and Cd6) points toward a pro-inflammatory and pro-fibrotic environment, providing a transcriptomic basis for the observed pulmonary pathology. In summary, the *Trpv4* mutation is associated with exacerbated lung fibrosis and significant alterations in the cellular landscape and pathological cell-cell communication, thereby revealing novel targets for anti-fibrotic intervention.

## Data Availability

The original contributions presented in the study are publicly available. This data can be found in the Gene Expression Omnibus (GEO) repository with the accession number GSE285127.
