# Supplementary figures and images for: Potential role of the Trpv4 c.1491+1G>A mutation in pulmonary fibrosis in a gene-edited mouse model
Source: Front Genet. 2026 Jun 18;17:1834091. doi: 10.3389/fgene.2026.1834091 (PMC13322678; doi:10.3389/fgene.2026.1834091)

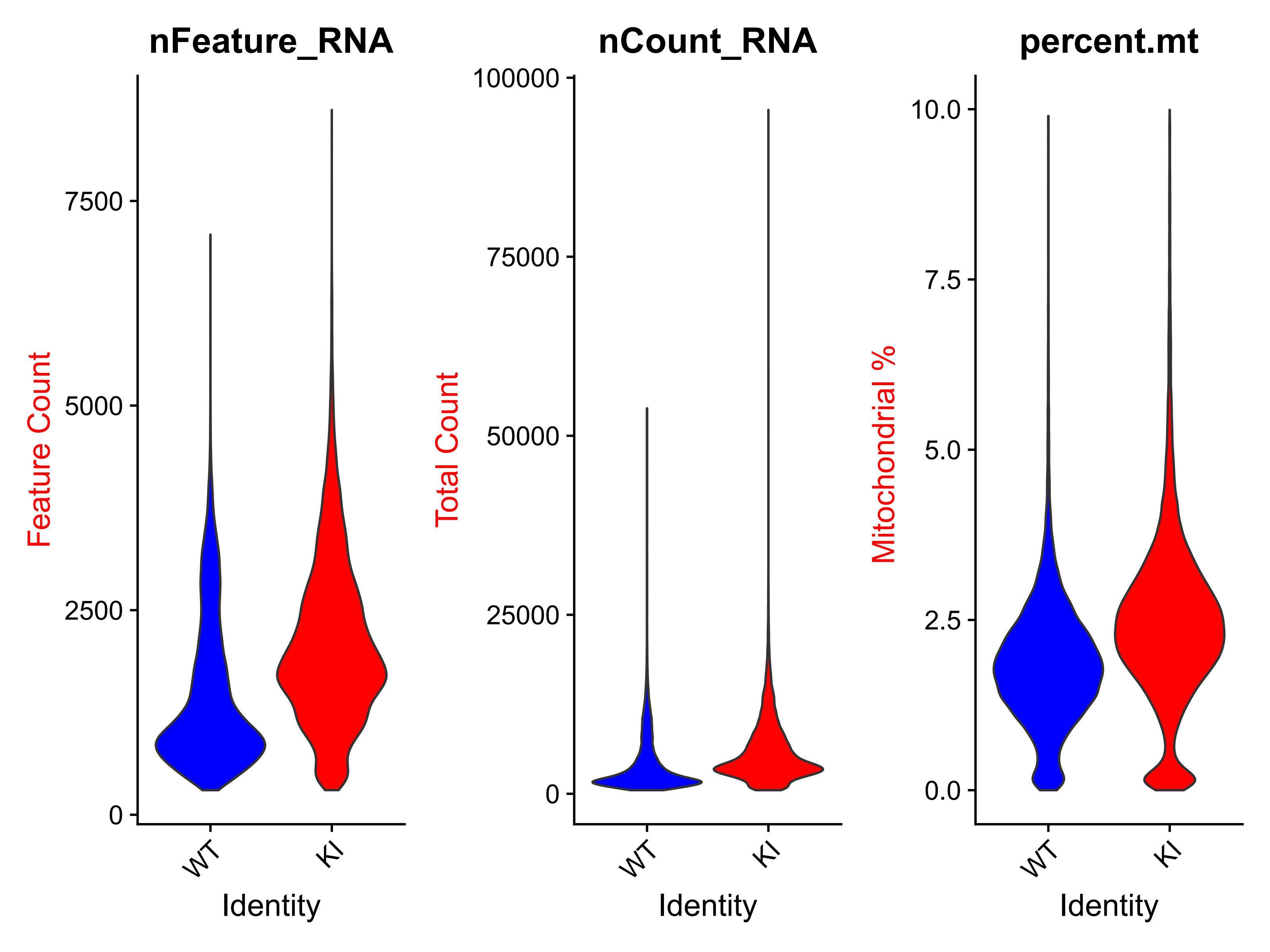

Supplement: Supplementary file 1 [file DataSheet3.zip › Supplementary_Figures/S1.jpg]

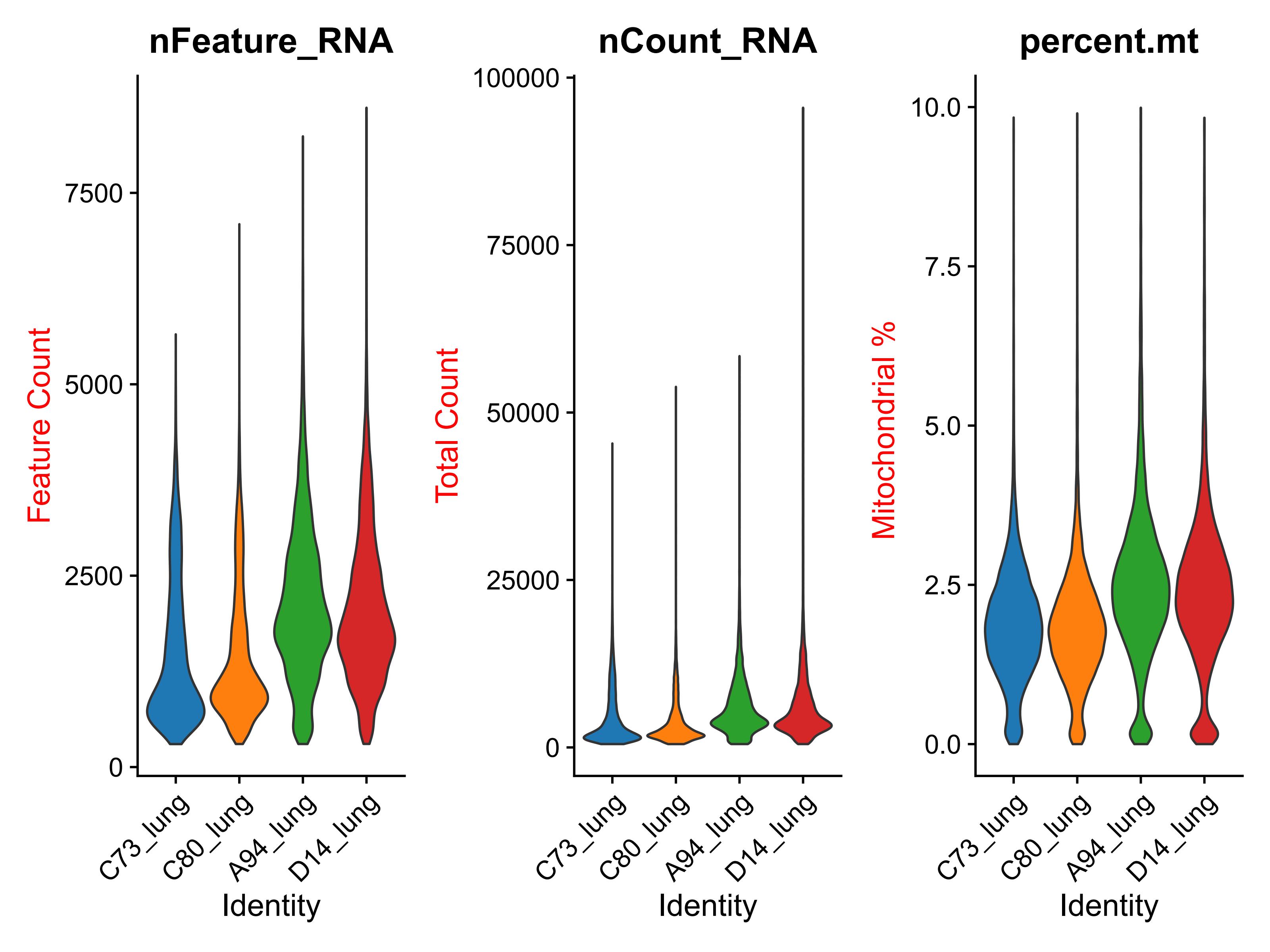

Supplement: Supplementary file 1 [file DataSheet3.zip › Supplementary_Figures/S2.jpg]

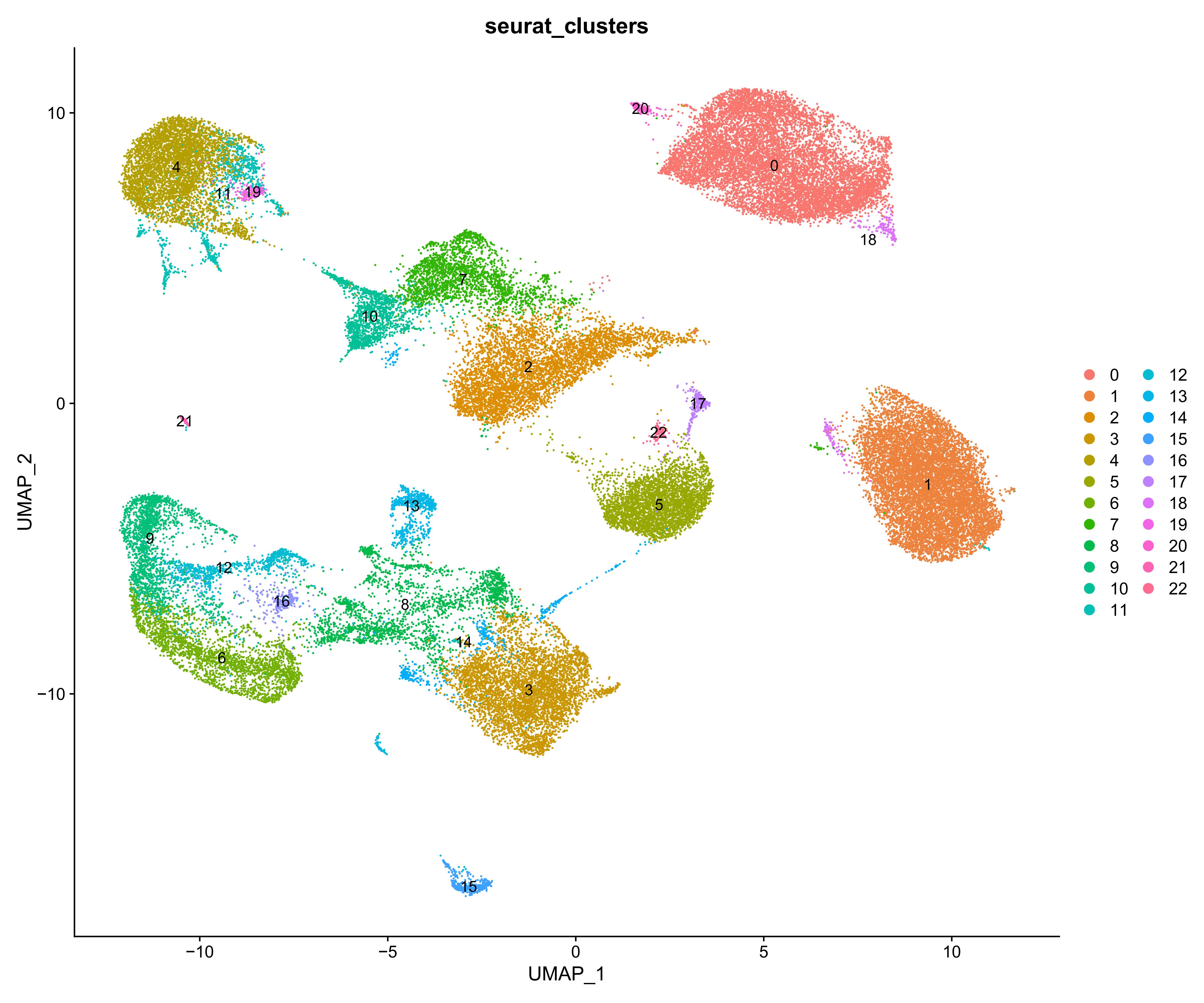

Supplement: Supplementary file 1 [file DataSheet3.zip › Supplementary_Figures/S4.jpg]

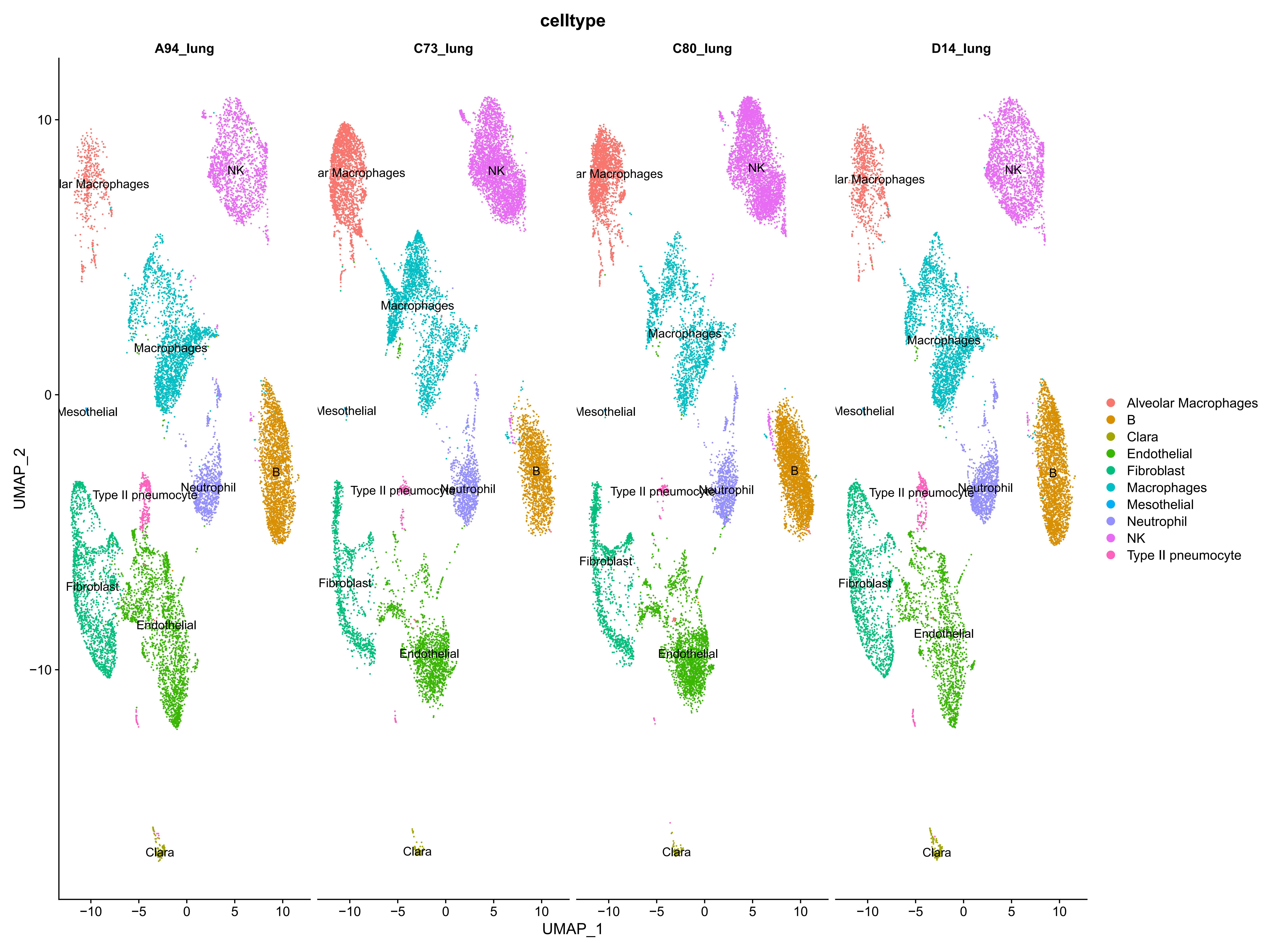

Supplement: Supplementary file 1 [file DataSheet3.zip › Supplementary_Figures/S5.jpg]

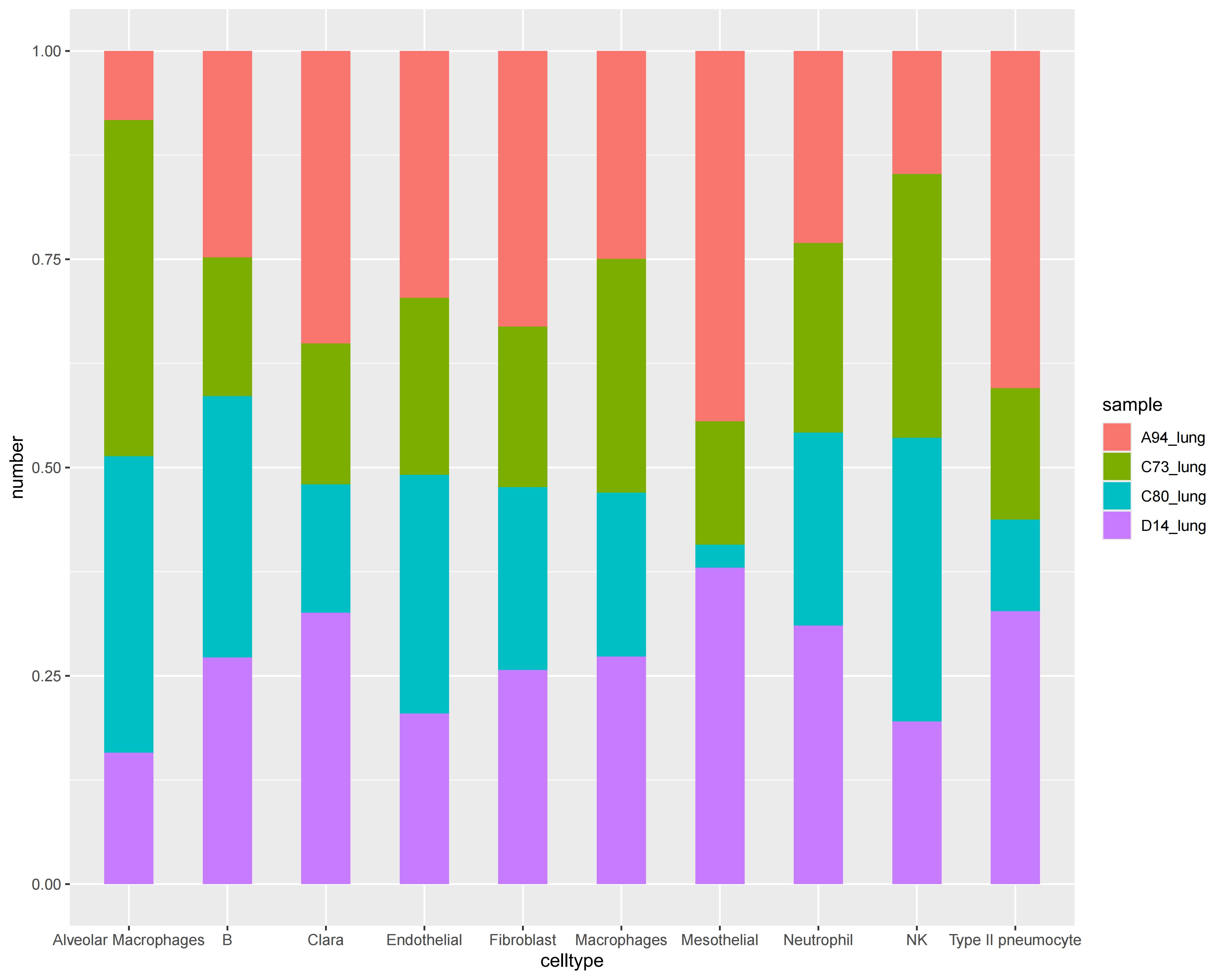

Supplement: Supplementary file 1 [file DataSheet3.zip › Supplementary_Figures/S6.jpg]

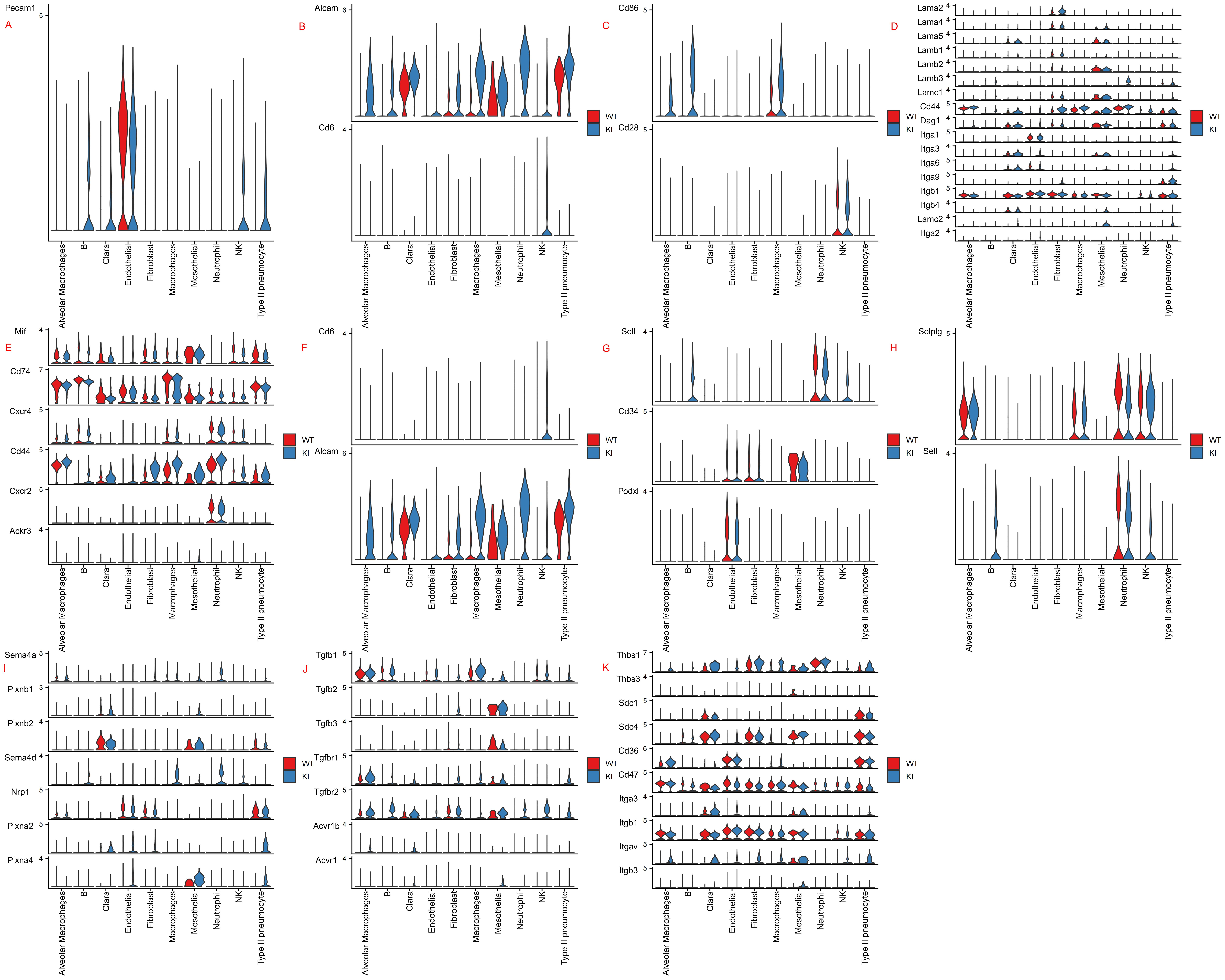

Supplement: Supplementary file 1 [file DataSheet3.zip › Supplementary_Figures/S7.jpg]

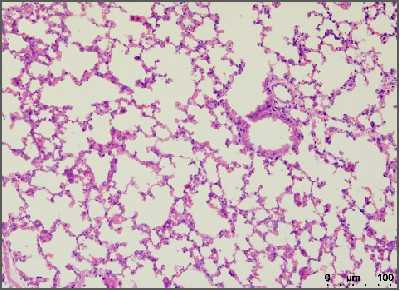

Supplement: Supplementary file 3 [file DataSheet1.zip › Supplementary.1/1.H&E+Masson/HE-WT.jpg]

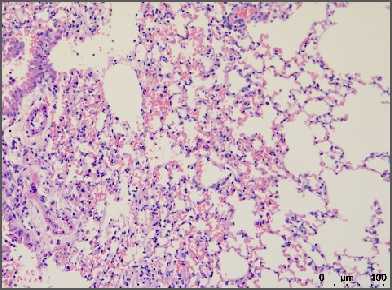

Supplement: Supplementary file 3 [file DataSheet1.zip › Supplementary.1/1.H&E+Masson/Het-HE.jpg]

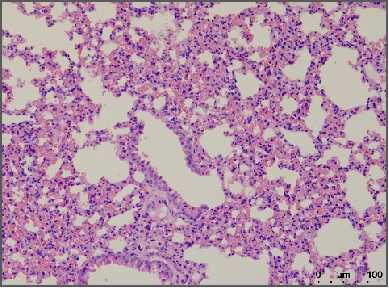

Supplement: Supplementary file 3 [file DataSheet1.zip › Supplementary.1/1.H&E+Masson/Hom-HE.jpg]

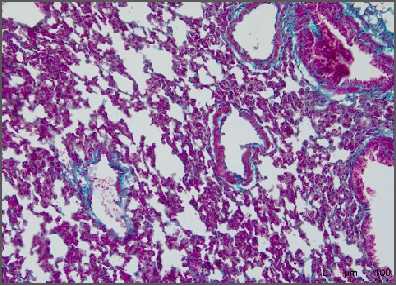

Supplement: Supplementary file 3 [file DataSheet1.zip › Supplementary.1/1.H&E+Masson/MS-Het.jpg]

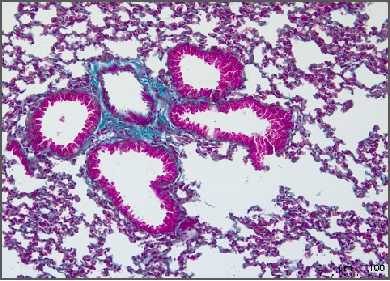

Supplement: Supplementary file 3 [file DataSheet1.zip › Supplementary.1/1.H&E+Masson/MS-Hom.jpg]

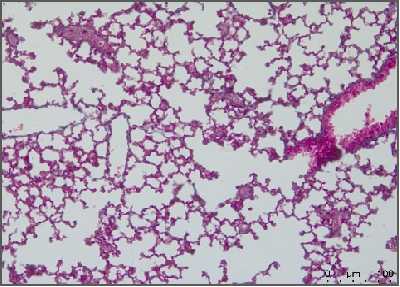

Supplement: Supplementary file 3 [file DataSheet1.zip › Supplementary.1/1.H&E+Masson/MS-WT.jpg]

relative mRNA level adjusted to  $\beta$ -actin

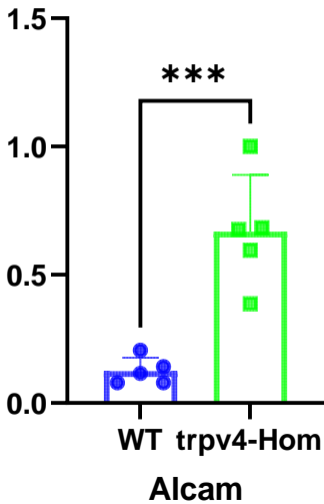

Supplement: Supplementary file 3 [file DataSheet1.zip › Supplementary.1/2.QPCR/Alcam qPCR.pdf]

relative mRNA level adjusted to  $\beta$ -actin

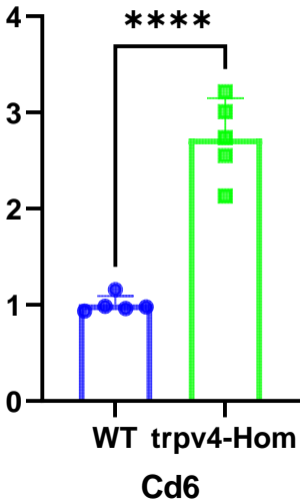

Supplement: Supplementary file 3 [file DataSheet1.zip › Supplementary.1/2.QPCR/Cd6 qpcr.pdf]

relative mRNA level adjusted to  $\beta$ -actin

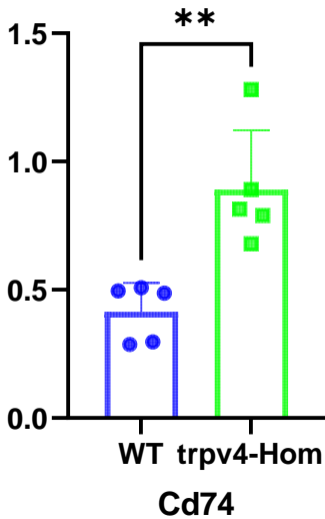

Supplement: Supplementary file 3 [file DataSheet1.zip › Supplementary.1/2.QPCR/Cd74 qpcr.pdf]

relative mRNA level adjusted to  $\beta$ -actin

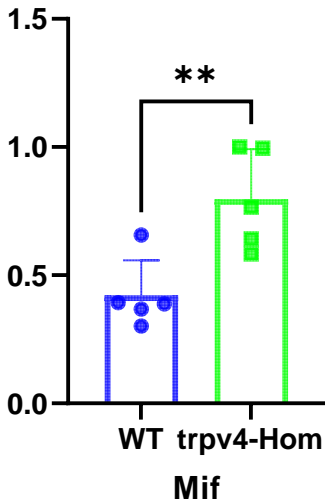

Supplement: Supplementary file 3 [file DataSheet1.zip › Supplementary.1/2.QPCR/Mif qPCR.pdf]

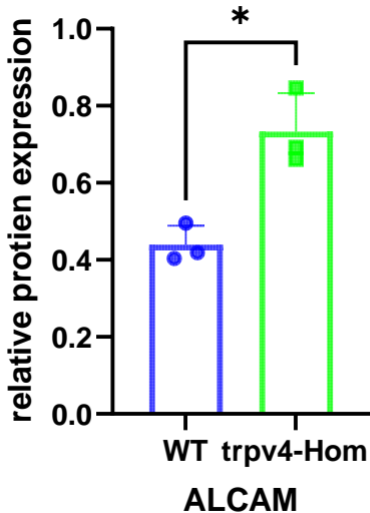

Supplement: Supplementary file 3 [file DataSheet1.zip › Supplementary.1/4.WB/ALCAM,CD74/ALCAM wb.pdf]

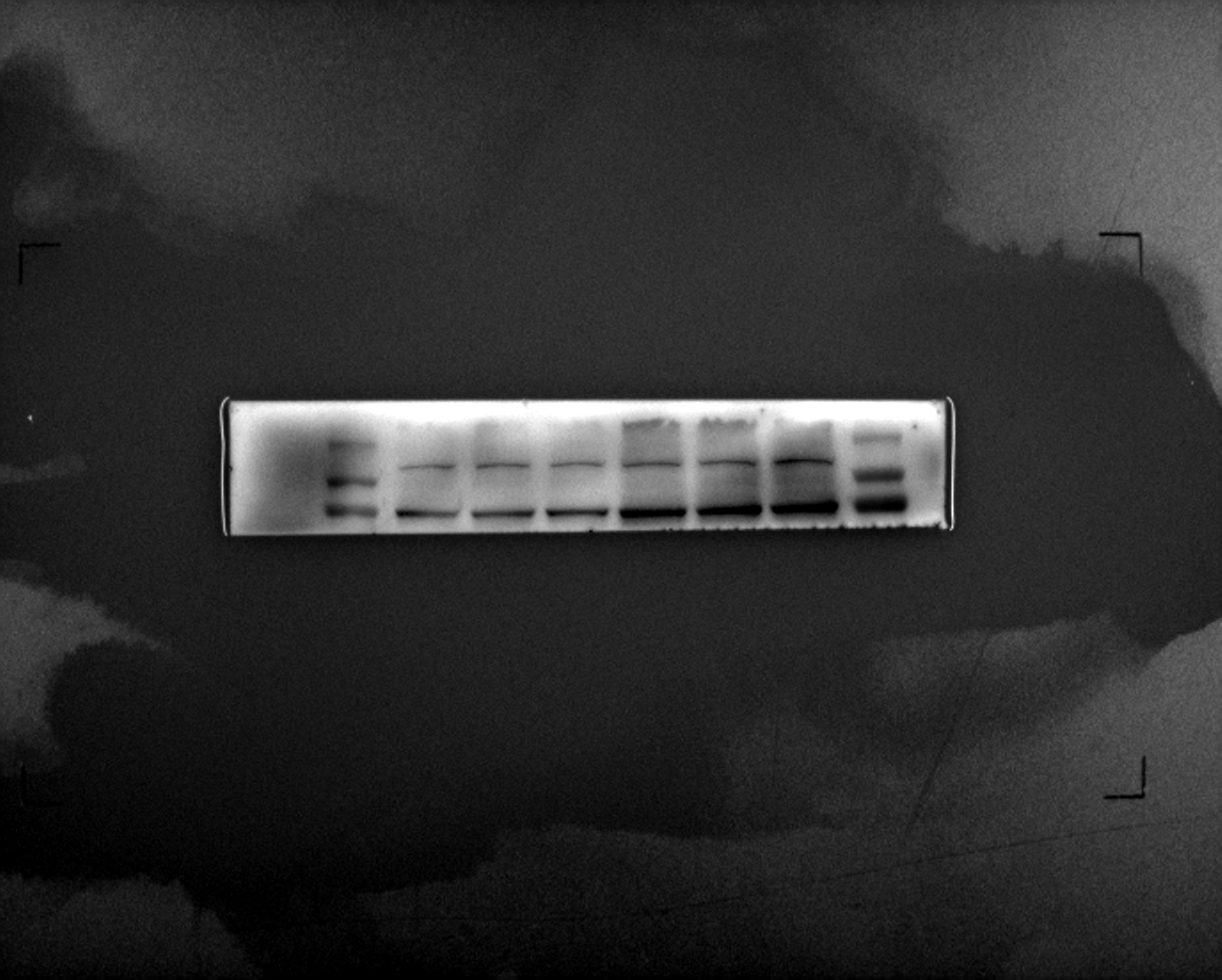

Supplement: Supplementary file 3 [file DataSheet1.zip › Supplementary.1/4.WB/ALCAM,CD74/ALCAM-1.Tif]

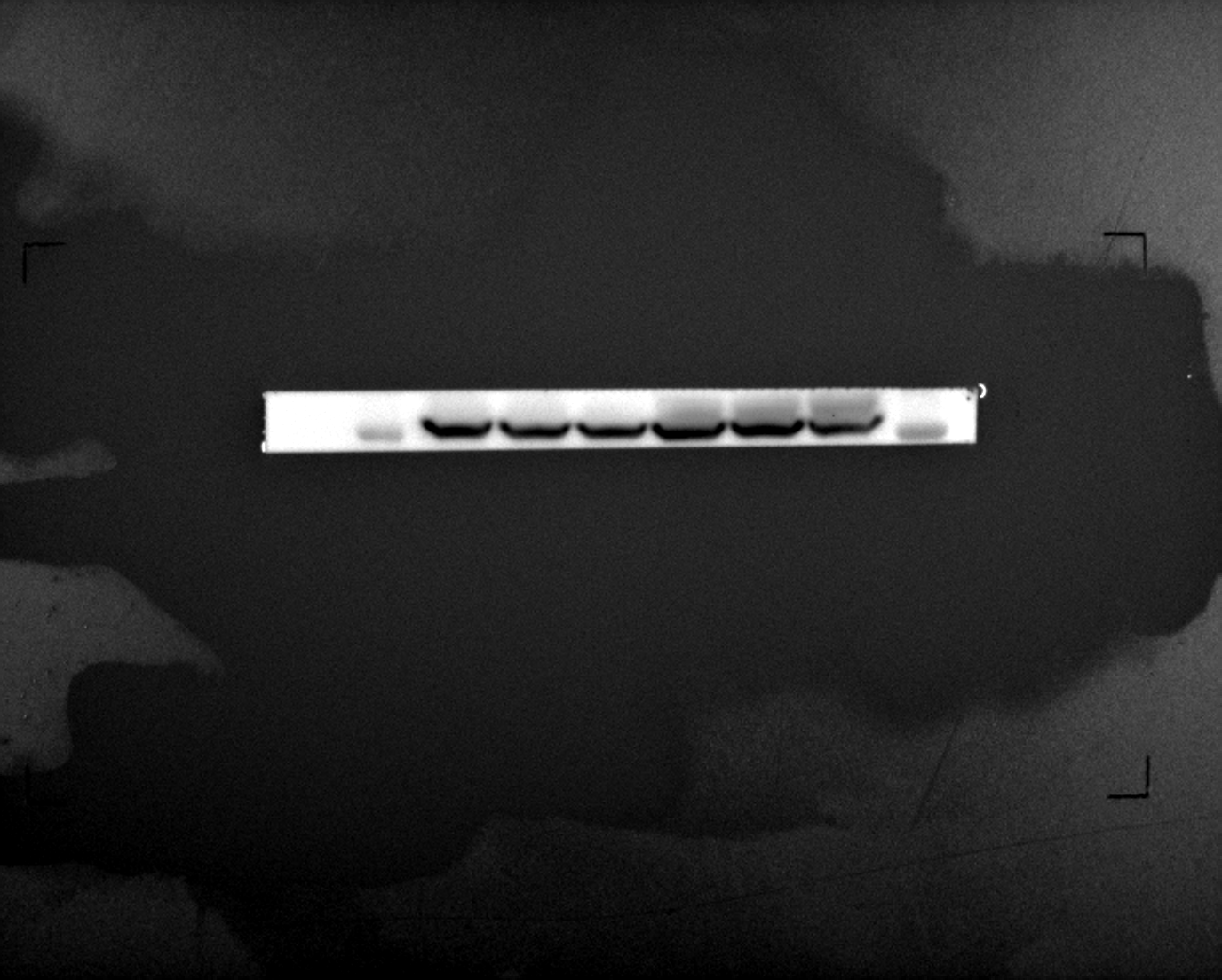

Supplement: Supplementary file 3 [file DataSheet1.zip › Supplementary.1/4.WB/ALCAM,CD74/B-actin-1.Tif]

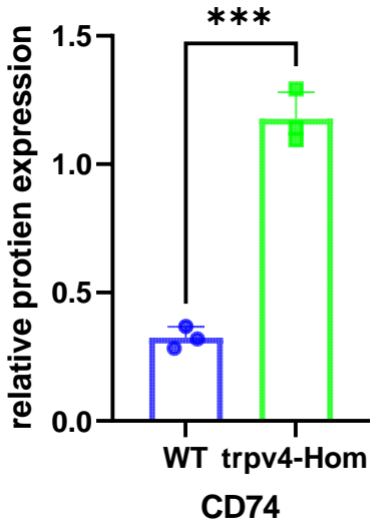

Supplement: Supplementary file 3 [file DataSheet1.zip › Supplementary.1/4.WB/ALCAM,CD74/CD74 wb.pdf]

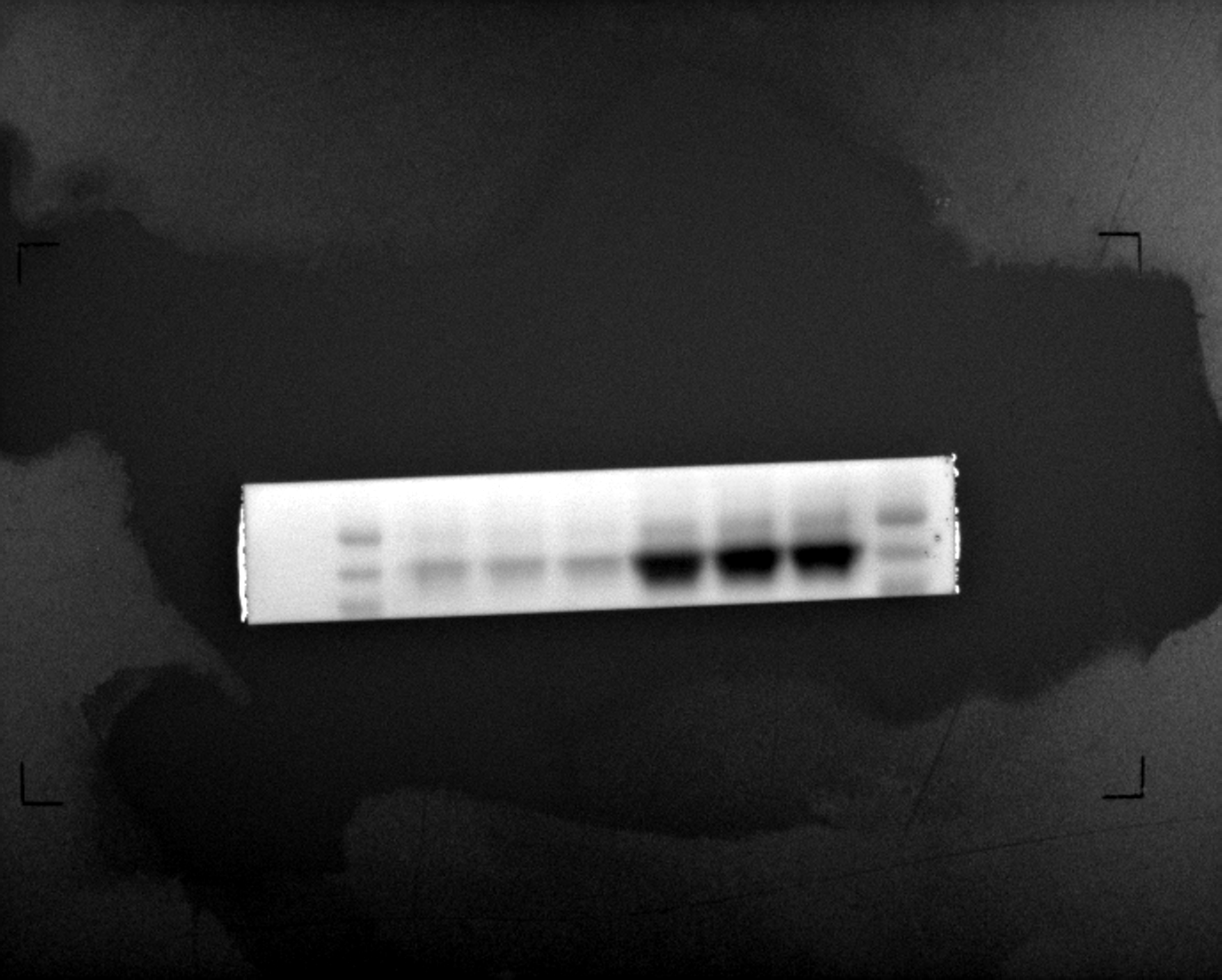

Supplement: Supplementary file 3 [file DataSheet1.zip › Supplementary.1/4.WB/ALCAM,CD74/CD74-1.Tif]

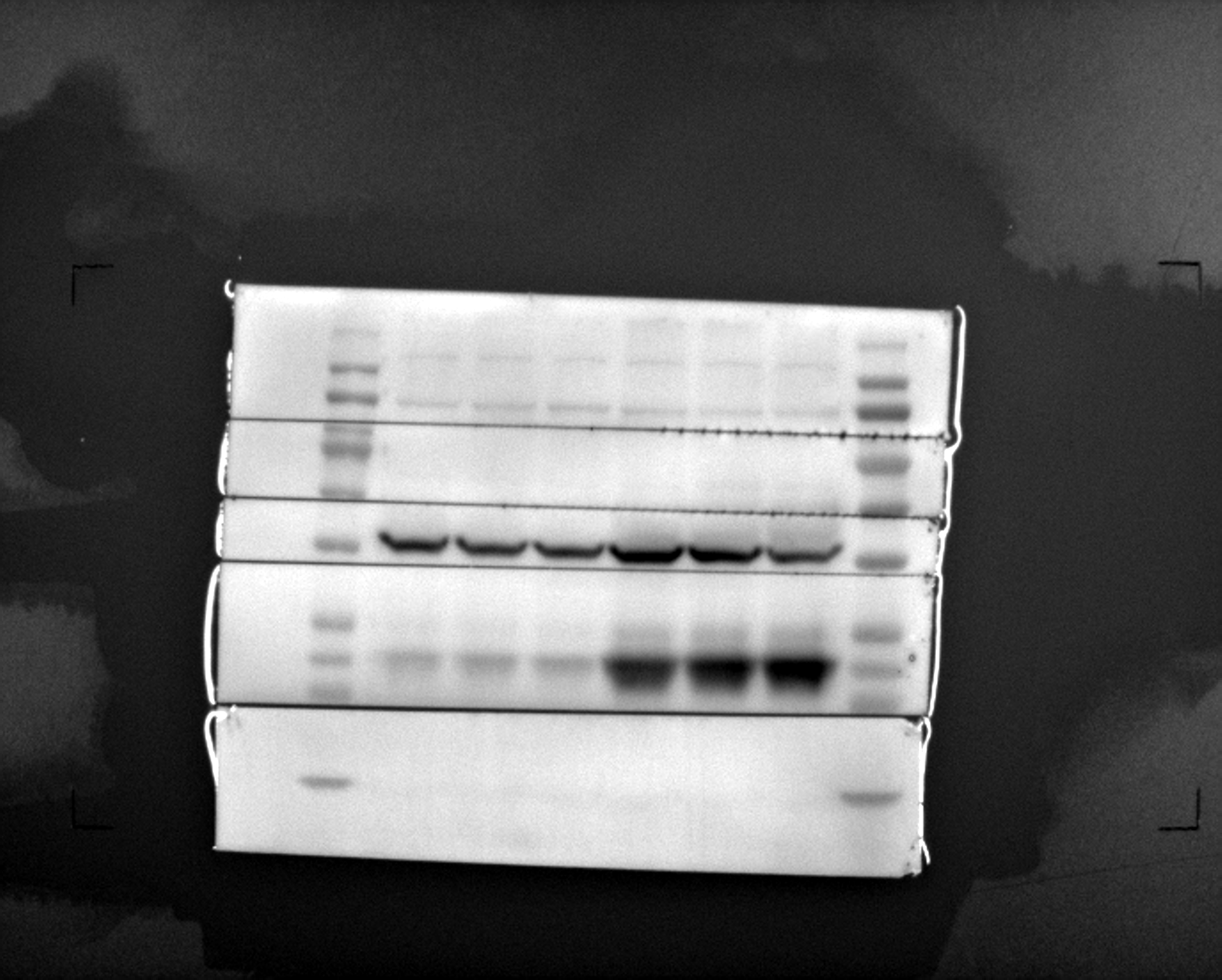

Supplement: Supplementary file 3 [file DataSheet1.zip › Supplementary.1/4.WB/ALCAM,CD74/Merge-1.1.Tif]

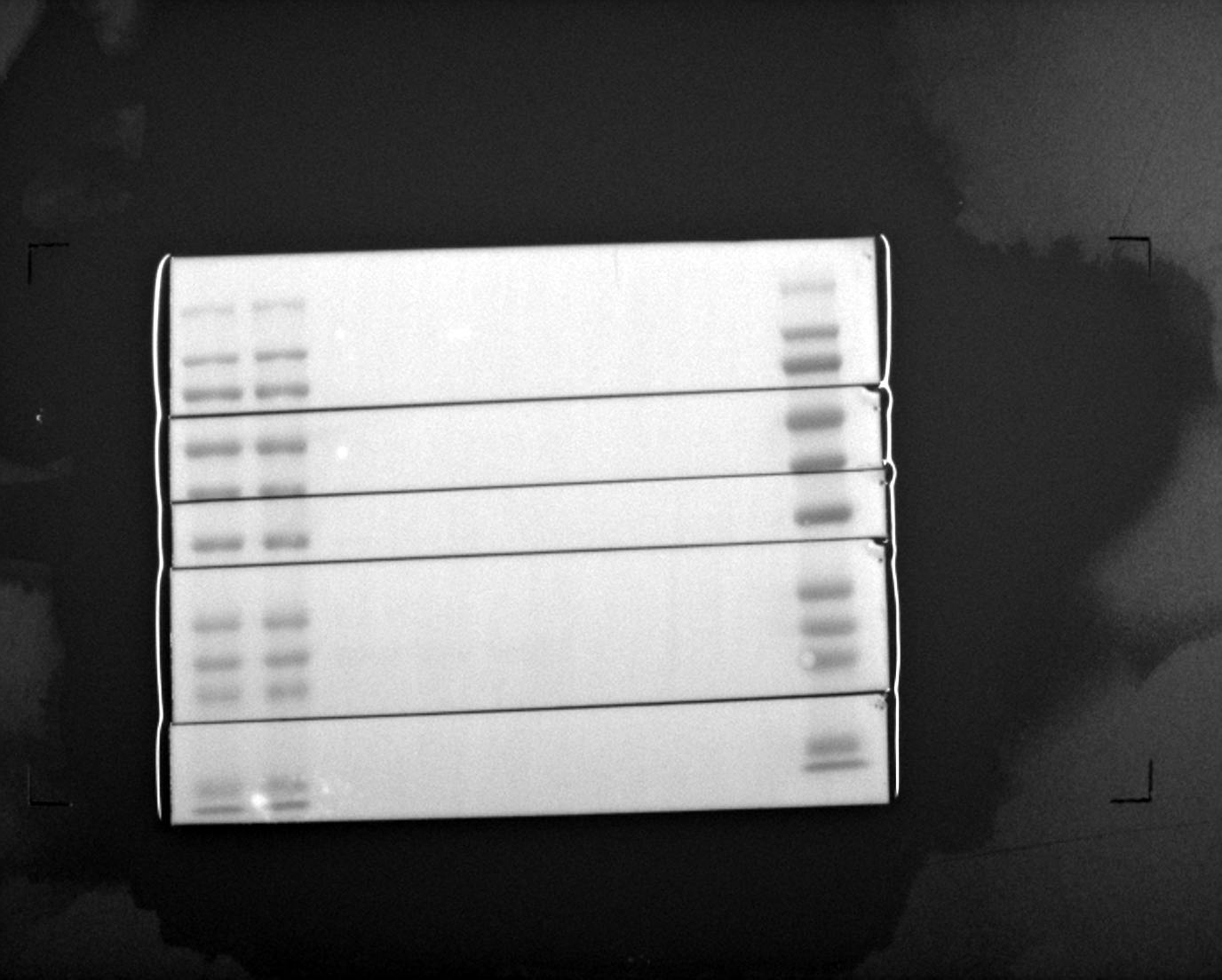

Supplement: Supplementary file 3 [file DataSheet1.zip › Supplementary.1/4.WB/ALCAM,CD74/Merge-2.Tif]

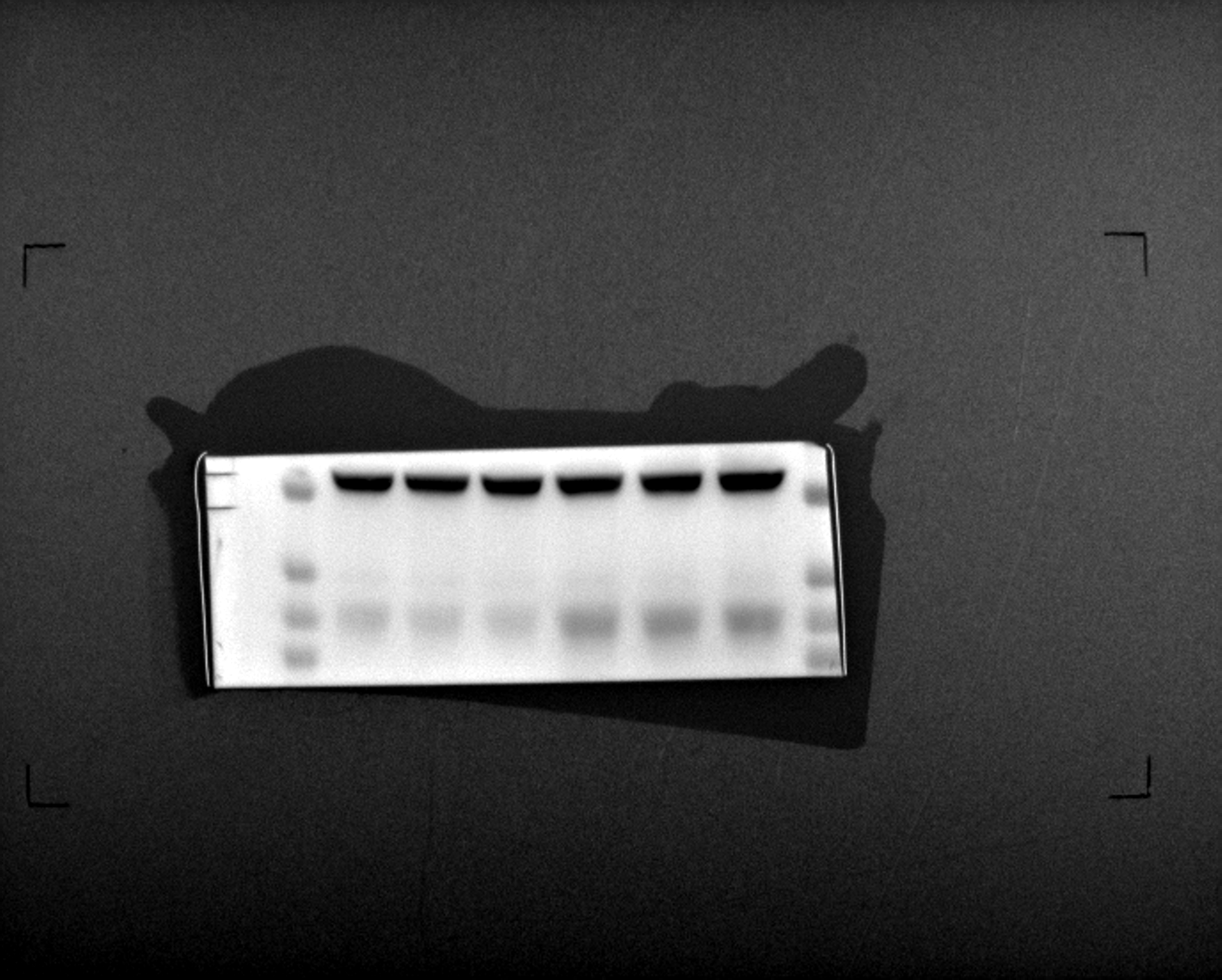

Supplement: Supplementary file 3 [file DataSheet1.zip › Supplementary.1/4.WB/CD6/b-actin.2-25.6.5.Tif]

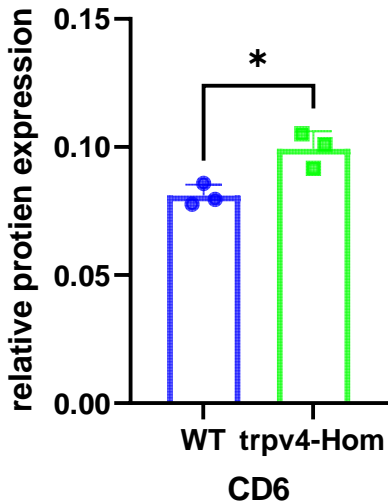

Supplement: Supplementary file 3 [file DataSheet1.zip › Supplementary.1/4.WB/CD6/CD6 wb.pdf]

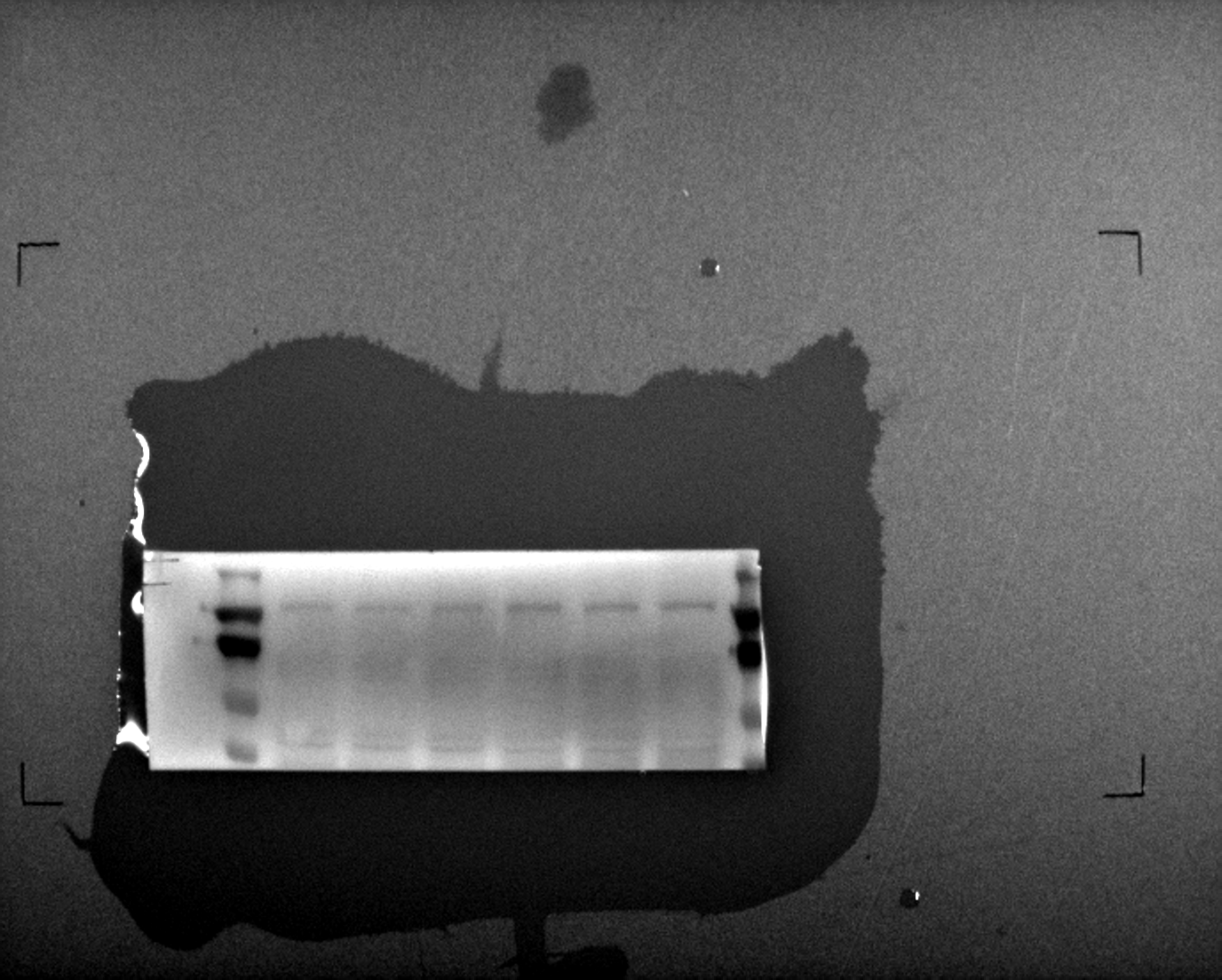

Supplement: Supplementary file 3 [file DataSheet1.zip › Supplementary.1/4.WB/CD6/Cd6-2-6.5.Tif]

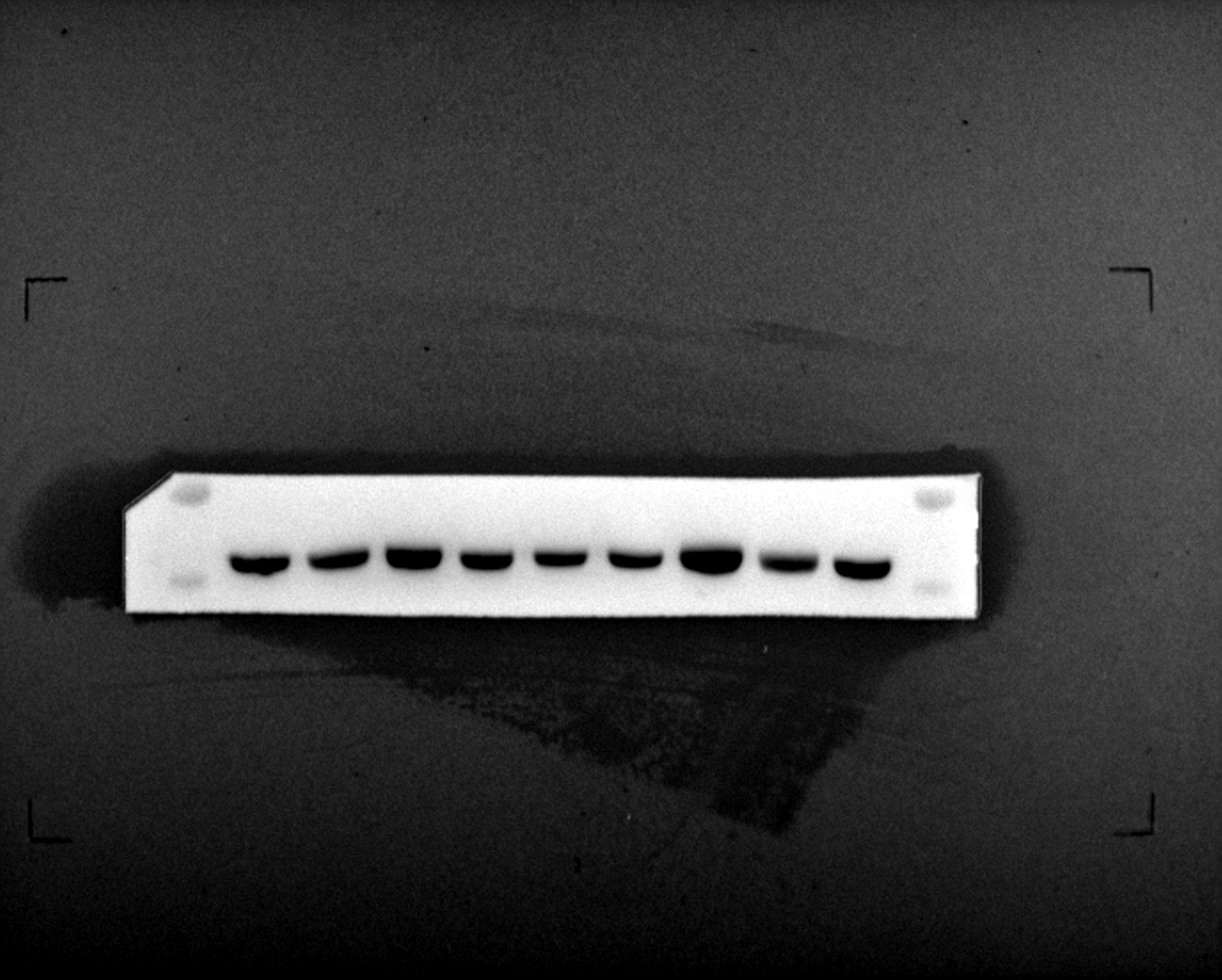

Supplement: Supplementary file 3 [file DataSheet1.zip › Supplementary.1/4.WB/Trpv4/Lung actin1 10.10.Tif]

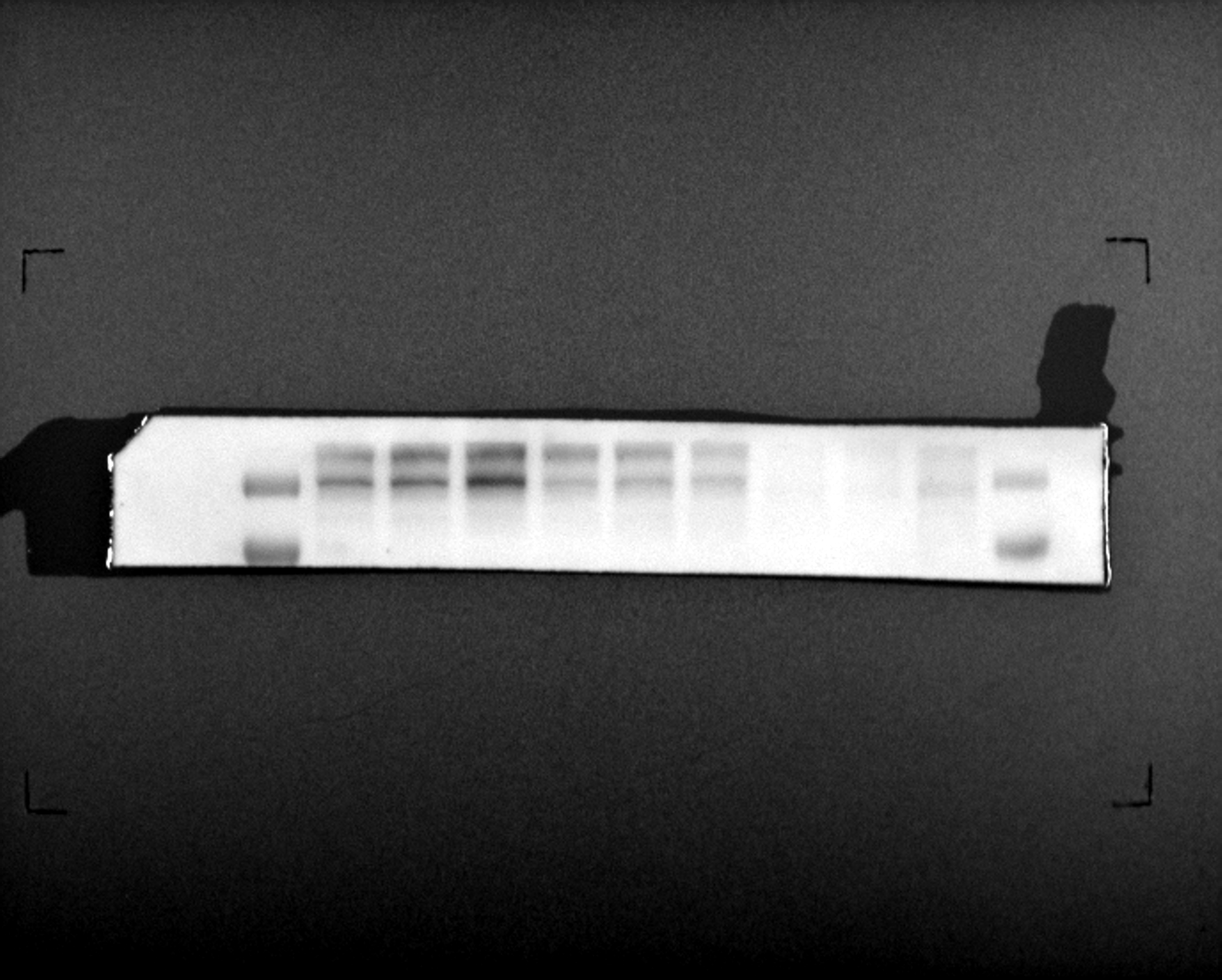

Supplement: Supplementary file 3 [file DataSheet1.zip › Supplementary.1/4.WB/Trpv4/Lung trpv4 1 10.11.Tif]

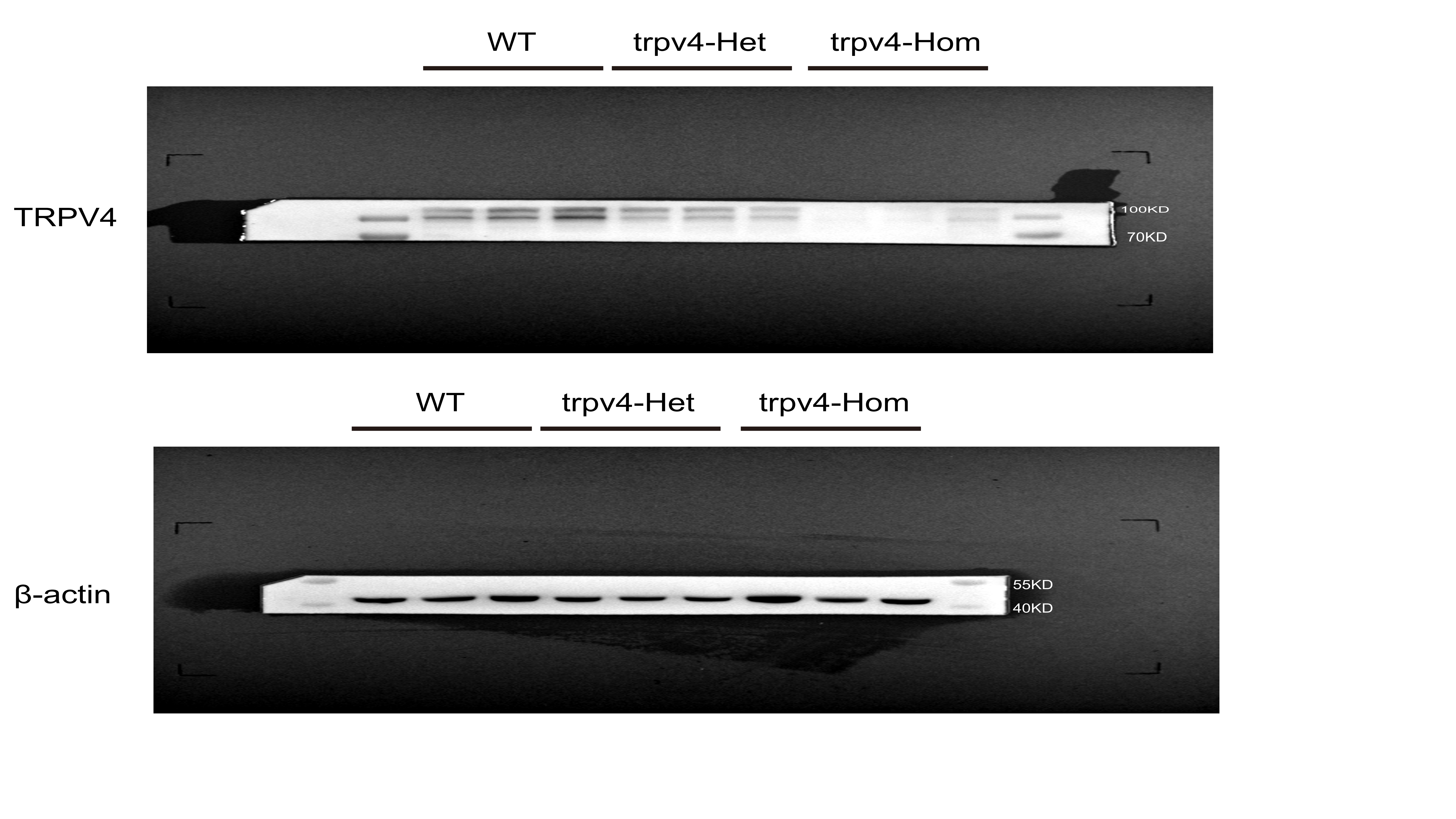

Supplement: Supplementary file 3 [file DataSheet1.zip › Supplementary.1/4.WB/Trpv4/Lung trpv41+actin1.png]

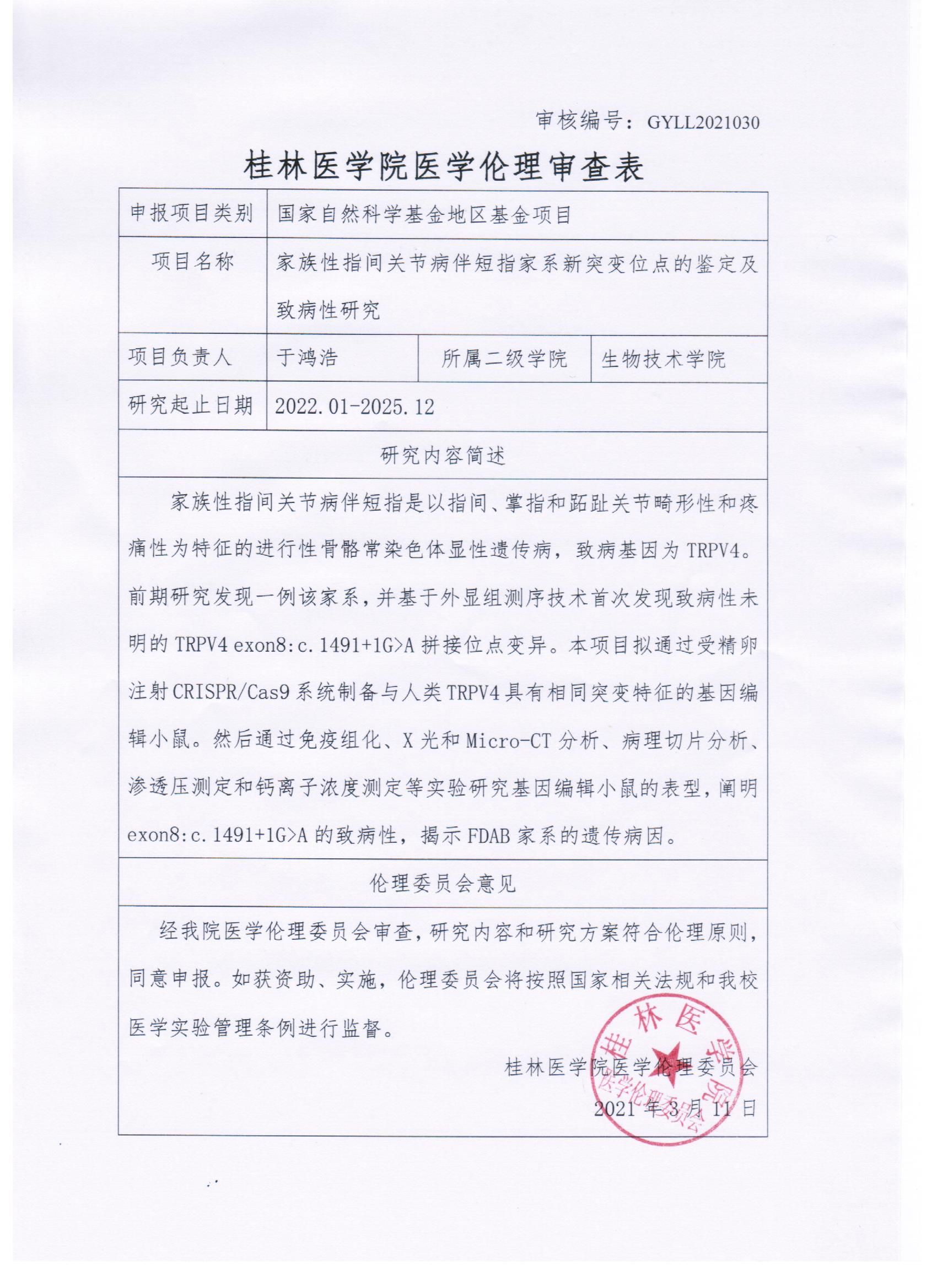

Supplement: Supplementary file 4 [file DataSheet2.zip › Supplementary.2/1.Ethical Document1.jpg]

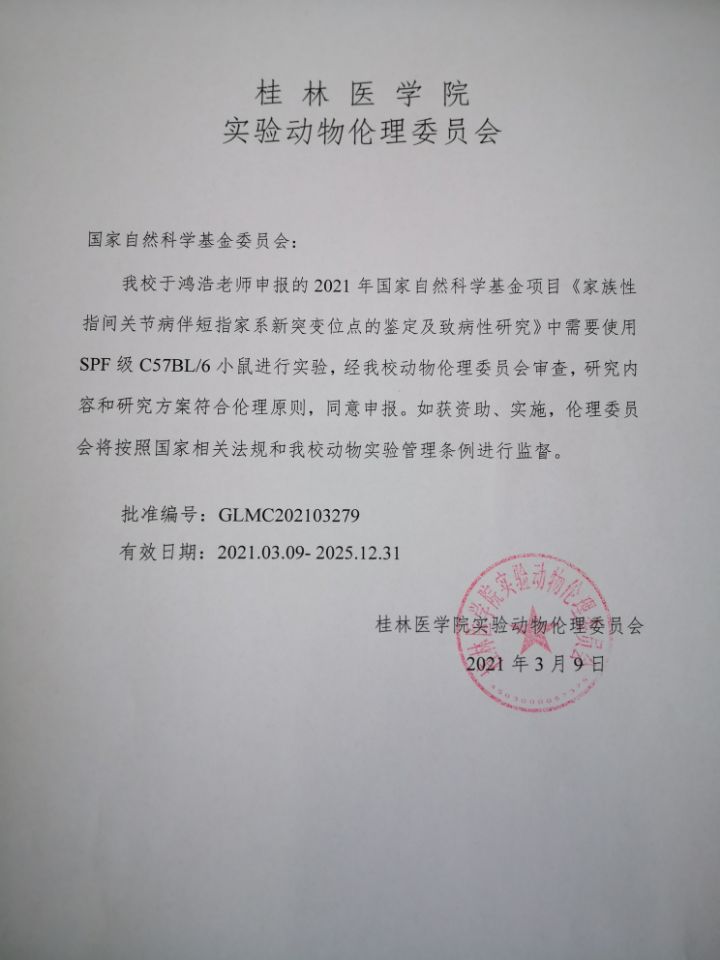

Supplement: Supplementary file 4 [file DataSheet2.zip › Supplementary.2/2.Ethical Document2.jpg]

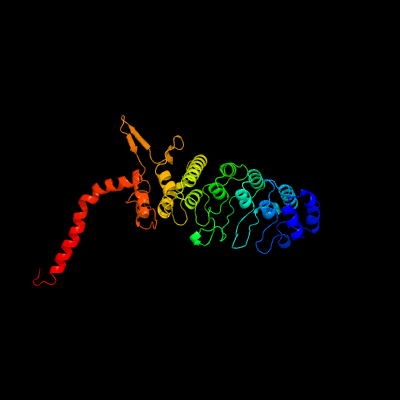

Supplement: Supplementary file 4 [file DataSheet2.zip › Supplementary.2/7.Three-dimensional structure1.png]

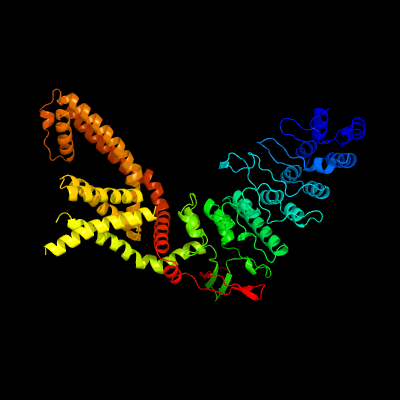

Supplement: Supplementary file 4 [file DataSheet2.zip › Supplementary.2/8.Three-dimensional structure2.png]
