# Supplementary material for: Potential role of the Trpv4 c.1491+1G>A mutation in pulmonary fibrosis in a gene-edited mouse model
Source: Front Genet. 2026 Jun 18;17:1834091. doi: 10.3389/fgene.2026.1834091 (PMC13322678; doi:10.3389/fgene.2026.1834091)
Supplement: Supplementary file 2 [file DataSheet4.pdf]

Supplementary Information Guide

Manuscript ID: 1834091

Title: Potential role of the Trpv4 c.1491+1G>A mutation in pulmonary fibrosis in a gene-edited mouse model

1. Overview of Supplementary Files

This study provides comprehensive raw data and supplementary materials organized into two main packages to support the findings presented in the main text.

Supplementary.1：

	File Name	Description	Supporting Section in Main Text
Supplementary.1	1.H&E+Masson	Original pathological section scans and high-magnification images of lung tissues from WT, Trpv4-Het and Trpv4-Hom mice	Section 2.6 & 3.2
	2.QPCR	The statistical analysis graph including Trpv4 and the validation genes (Mif, Cd74, Alcam, Cd6).	Section 2.5 & 3.8
	3.R	R language source code.	Section 2.10
	4.WB	The original full-exposure band images and quantitative analysis data of the TRPV4 protein as well as the various validation indicator proteins (ALCAM, CD6, CD74) are included.	Section 2.5 & 3.8
Supplementary.2	1.Ethical Document 1	Guilin Medical University Medical Ethics Review Form	Section 2.1
	2.Ethical Document 2	Guilin Medical University Laboratory Animal Ethics Committee	Section 2.1
	3.Table S1	PCR primer sequences used for genotyping and expression analysis.	Section2.4
	4.Table S2	qPCR primer sequences used for genotyping and expression analysis.	Section2.5
	5.Table S3	Full CDS and predicted protein sequences for WT and Trpv4 c.1491+1G>A mutant mod	Section3.2

		els.	
	6.Table S4	Comprehensive list of differentially expressed genes (DEGs) across all 10 annotated cell types.	Section3.3
	7.Three-dimensional structure1	High-resolution 3D protein structure modeling files for truncated TRPV4.	Section3.2
	8.Three-dimensional structure2	High-resolution 3D protein structure modeling files for WT.	Section3.2
	9.Trpv4 CDS Sequence-Editing	The complete Coding DNA Sequence (CDS) of the Trpv4 gene in the gene-edited model, showing the splicing-induced changes.	Section3.2
	10.Trpv4 CDS Sequence-WT	The complete wild-type (WT) Trpv4 Coding DNA Sequence used as a reference for mutation analysis.	Section3.2
	11.Trpv4 primer sequences		Section3.2
	12.TRPV4 Protein Sequence-Editing	The predicted amino acid sequence of the truncated TRPV4 protein resulting from the c.1491+1G>A mutation.	Section3.2
	13.TRPV4 Protein Sequence-WT	The standard wild-type TRPV4 amino acid sequence used for structural and functional comparison.	Section3.2
	14.Zhou Yujiao Genetic Testing Report	Clinical report of whole exome sequencing (WES) for the proband and detailed analysis results of genetic variations.	Section2.2
	15.Zhou Yujiao Family Genetic Testing Report	The Sanger sequencing verification report and genetic map summary of the members of this FDAB family.	Section2.2
Supplementary_Figures	S1	The violin plot shows the data quality of WT and TRPV4 *Trpv4*-Hom mouse.	Section3.3
	S2	The violin plot shows the data sample quality of WT and *Trpv4*-Hom mouse.	Section3.3

	S3	The heat map displays the differentially expressed genes for each sample.	Section3.3
	S4	Display UMAP graphs for all clusters.	Section3.3
	S5	Comparison of the distribution of major cell clusters between the WT group and the *Trpv4*-Hom group.	Section3.3
	S6	A histogram that describes the proportional changes of each cell type in a single sample.	Section3.3
	S7	The violin plot (A-K) shows the distribution of genes related to the differential pathways (PECAM1,ALCAM,CD86,LAMININ,MIF,CD6,SELL,SELPLG,SEMA4,TGF,THBS)between B cells and NK cells.	Section3.6
