# Supplementary material for: Potential role of the Trpv4 c.1491+1G>A mutation in pulmonary fibrosis in a gene-edited mouse model
Source: Front Genet. 2026 Jun 18;17:1834091. doi: 10.3389/fgene.2026.1834091 (PMC13322678; doi:10.3389/fgene.2026.1834091)
Supplement: Supplementary file 4 [file DataSheet2.zip › Supplementary.2/11.Trpv4 primer sequences.docx]

Supplementary Table 1. PCR amplification of primer sequences

| Name | Sequences (5'-3') |
| --- | --- |
| Trpv4-M-F | TCTTGGCTGCCCAGAGTA |
| Trpv4-M-R | CCCAAACATCTGCGTCCT |

Supplementary Table 2. QPCR primer sequences for *Trpv4* mice

| Name | Sequences (5'-3') |
| --- | --- |
| Trpv4-Q-F | GAGACAAGTGGCGTAAGTT |
| Trpv4-Q-R | TCCTGTGAAGAGCGTGAT |
