# Supplementary material for: Potential role of the Trpv4 c.1491+1G>A mutation in pulmonary fibrosis in a gene-edited mouse model
Source: Front Genet. 2026 Jun 18;17:1834091. doi: 10.3389/fgene.2026.1834091 (PMC13322678; doi:10.3389/fgene.2026.1834091)
Supplement: Supplementary file 4 [file DataSheet2.zip › Supplementary.2/14.Zhou Yujiao Genetic Testing Report.pdf]

遗传性疾病基因检测报告（再分析）

样本信息

样本编号	检测类型	送检单位	报告日期
20Y01164	再分析	桂林医学院附属医院	2020-06-10

受检者信息

姓名	与受检者关系	检测项目	性别	年龄	样本类型	送检日期
周玉姣	受检者	WES	女	34周岁	外周血	2020-05-27

临床信息

临床表现与怀疑疾病：结缔组织病？多发性动脉炎？本人10岁时，因“发热”导致双手指端部分关节萎缩脱落（左手食指，右手大拇指，小指）；其女儿出生3天时，因“发热”导致右手无名指末端萎缩脱落2个指节；遗传性皮肤病panel（康旭）未见明显异常。其余家系成员健康情形未见此家系图中。

根据提供的临床资料提取用于报告解读的关键词为：结缔组织病、多发性动脉炎、动脉炎、关节萎缩、手指自发性离断、发热

新增临床信息

女儿加做母亲同一提示位点的一代验证，检测结果为杂合突变，能否提升致病性证据？

检测方法

本检测使用全外显子组高通量测序检测技术，利用贝瑞基因自主研发的Verita Trekker®变异位点检测系统和Enliven®变异位点注释解读系统对数据进行分析。

检测结果

受检者样本未检测到与本案例表型相关的致病性SNV、InDel变异，另外有1个提示关注位点。

检测人：冯宇　　审核人：[illegible]

北京贝瑞和康医学检验实验室有限公司 报告专用章 1101051881537

核心报告内容

核心结果解读

一．结果

受检者样本未检测到与本案例表型相关的致病性SNV、InDel变异，另外有1个提示关注位点。

二．SNV、InDel结果

根据美国医学遗传学与基因组学学会(ACMG)指南(Richards et al., 2015)，以及对HPO、OMIM、GHR等公共数据库查找与结缔组织病、多发性动脉炎、关节萎缩、手指自发性离断、发热、指端断离等表型或疾病相关基因变异进行筛查，在受检者样本中未检测到与本案例表型相关的可能致病的SNV、InDel变异。

以上结果请结合家系和临床进一步分析。

提示关注内容

SNV及InDel检测结果

基因	突变位置	外显子	HGVS	突变类型	杂合性	变异评级	疾病及遗传方式	提示说明
TRPV4	chr12:109794328-109794328	exon8	NM_021625.4:c.1491+1G>A:.	splicing	先证者：杂合<br>女儿：杂合	VUS	Digital arthropathy-brachydactyly, familial（家族性指间关节病伴短指）, AD	表型部分相符，与AD遗传模式相符，临床意义未明

・表示数据库无收录。参考数据库版本为：Human Genome 38 (hg38/GRCh38)。

更多位点信息请临床医生参照“Cruxome系统”。

注：该提示关注报告内容是根据美国医学遗传学与基因组学学会(ACMG)指南(Richards et al., 2015)，经遗传模式、发病年龄、人群频率、危害预测过滤等，选择与表型相关但尚未有充分证据证明致病性的变异报出，结果仅供临床参考。更多更全面的临床表型信息、父母及家系其他成员的基因检测补充证据以及文献或数据库的更新均有助于进一步明确变异的致病性。

提示关注结果解读

上述位点提示关注原因为基于现有的信息判断临床致病性证据不足，但均与本案例表型相关。

根据美国医学遗传学与基因组学学会(ACMG)指南(Richards et al., 2015)，提示基因TRPV4的c.1491+1G>A突变为一个意义未明突变位点，与常染色体显性遗传疾病Digital arthropathy-brachydactyly, familial（家族性指间关节病伴短指）相关。

1）TRPV4:NM_021625.4:exon8:c.1491+1G>A:.变异：意义未明（PM2）

中等致病性证据PM2：该变异在贝瑞基因中国人群特有数据库“神州基因组数据库”、人类外显子数据库(ExAC)、参考人群千人基因组(1000G)和人群基因组突变频率数据库(gnomAD)中没有发现。

疾病特征与本案例表型相符度：部分相符

经公共数据库查询，基因TRPV4(OMIM:605427)的突变会导致常染色体显性遗传疾病Digital arthropathy-brachydactyly, familial（家族性指间关节病伴短指）(OMIM:606835, Lamande et al., 2011)。Digital arthropathy-brachydactyly, familial（家族性指间关节病伴短指）主要表现为：中末节指骨进行性短指、指间关节和掌指关节的进行性关节病、远节趾骨短小、关节病、中节趾骨短、手指短中节指骨、指（趾）中远端不成比例地缩短、指桡侧偏斜。

综上所述，建议临床补充更多更全面的表型信息、家系其他成员的基因检测结果等，再进一步评估变异位点与本案例的相关性。

一代验证结果信息

TRPV4:NM_021625.4:exon8:c.1491+1G>A:.

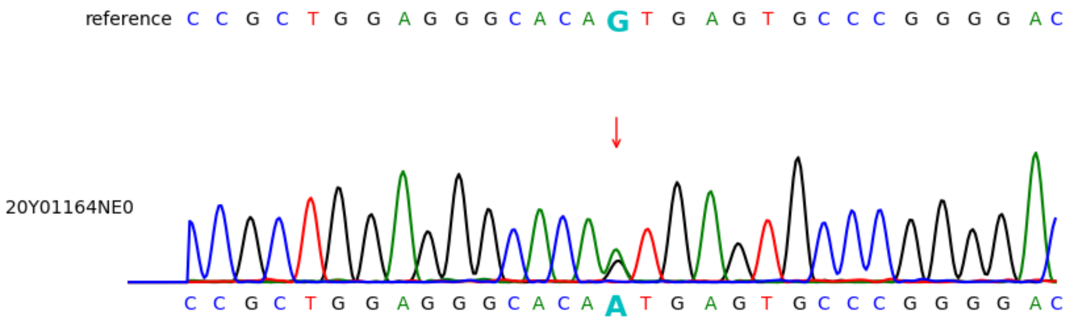

图注: 20Y01164NE0:女儿

SNV及InDel扩展报告内容

该扩展报告内容为ACMG SF v2.0 (Kalia et al., 2017)推荐报导的59个基因，经Enliven®变异位点注释解读系统注释，对遗传模式、人群频率过滤，未发现致病及可能致病性位点，结果仅供临床参考。

59个基因列表

ACTA2	*ACTC1*	*APC*	*APOB*	*ATP7B*	*BMPR1A*	*BRCA1*	*BRCA2*
CACNA1S	*COL3A1*	*DSC2*	*DSG2*	*DSP*	*FBN1*	*GLA*	*KCNH2*
KCNQ1	*LDLR*	*LMNA*	*MEN1*	*MLH1*	*MSH2*	*MSH6*	*MUTYH*
MYBPC3	*MYH11*	*MYH7*	*MYL2*	*MYL3*	*NF2*	*OTC*	*PCSK9*
PKP2	*PMS2*	*PRKAG2*	*PTEN*	*RB1*	*RET*	*RYR1*	*RYR2*
SCN5A	*SDHAF2*	*SDHB*	*SDHC*	*SDHD*	*SMAD3*	*SMAD4*	*STK11*
TGFBR1	*TGFBR2*	*TMEM43*	*TNNI3*	*TNNT2*	*TP53*	*TPM1*	*TSC1*
TSC2	*VHL*	*WT1*					

声明

1. 本报告结果只对本次送检样品负责，检测结果仅供临床参考。
2. 本检测主要针对全外显子组区域及剪切边界5bp范围内的单碱基变异(SNVs)和外显子区域50bp以内的插入/缺失(InDels)。
3. 本检测不包含poly结构、串联重复序列、富含GC区域以及存在同源相似序列（假基因），同时对50bp以上的插入/缺失(InDels)存在一定的局限性。
4. 由于全外显子组捕获的技术局限性，目前全外显子组检测不能保证覆盖100%的外显子区域。
5. 报告结果不能排除因母源污染造成的影响以及低比例嵌合的可能性，结果仅供临床参考。
6. 本检测阴性结果不排除由单亲二倍体UPD；染色体平衡易位、倒位、环状；生殖细胞嵌合；表观遗传学；多基因病及其他非遗传因素（感染、药物、辐射等环境因素）引起的可能性。
7. 检测数据分析仅依据医生或患者提供的临床信息，同时变异解读受制于文献和数据库的及时性，所报告的变异与临床疾病相关性需要由临床医生结合患者表型综合分析。
8. 限于现有医疗技术与诊断水平难以确诊，医师提供遗传咨询意见，育龄夫妇可以选择避孕、节育、不孕等相应的医学措施。

附录一：测序参数

样本	测序项目	20X覆盖度
受检者	人类全外显子组	98.65%

附录二：生信分析方法及数据库

变异位点致病性评级及数据解读规则参考美国医学遗传学与基因组学学会(ACMG)指南(Richards et al., 2015)，排除千人基因组、ExAC、gnomAD、贝瑞基因中国人群特有数据库“神州基因组数据库”等数据库中突变频率大于1%的变异位点，去除非功能性变异位点（如同义突变、非编码区突变等），再经过致病性预测（SIFT、Polyphen2、CADD等软件）、临床症状对照、相关疾病数据库查询与文献参考等综合考虑，找到候选基因变异位点进行家系验证。所用注释数据库为： Human Genome 38 (hg38/GRCh38)、RefSeq、dbSNP150、1000 Genomes phase3、ExAC10、gnomAD r2.1.1、贝瑞基因中国人群特有数据库“神州基因组数据库”等数据库，所用解读数据库包括DGV、DECIPHER、OMIM、UCSC、ClinVar、HGMD及PubMed等数据库。

参考文献

Richards S, Aziz N, Bale S, et al. Standards and Guidelines for the Interpretation of Sequence Variants: A Joint Consensus Recommendation of the American College of Medical Genetics and Genomics and the Association for Molecular Pathology[J]. Genetics in Medicine Official Journal of the American College of Medical Genetics, 2015, 17(5):405.

Kalia S S, Adelman K, Bale S J, et al. Recommendations for reporting of secondary findings in clinical exome and genome sequencing, 2016 update (ACMG SF v2.0): a policy statement of the American College of Medical Genetics and Genomics[J]. Genetics in Medicine Official Journal of the American College of Medical Genetics, 2017, 19(2):249.

Lamande S R, Yuan Y, Gresshoff I, et al. Mutations in TRPV4 cause an inherited arthropathy of hands and feet[J]. Nature Genetics, 2011, 43(11):1142-1146.

Kearney H M, Thorland E C, Brown K K, et al. American College of Medical Genetics standards and guidelines for interpretation and reporting of postnatal constitutional copy number variants[J]. Genetics in Medicine Official Journal of the American College of Medical Genetics, 2011, 13(7):680-5.
