# Supplementary material for: Potential role of the Trpv4 c.1491+1G>A mutation in pulmonary fibrosis in a gene-edited mouse model
Source: Front Genet. 2026 Jun 18;17:1834091. doi: 10.3389/fgene.2026.1834091 (PMC13322678; doi:10.3389/fgene.2026.1834091)
Supplement: Supplementary file 4 [file DataSheet2.zip › Supplementary.2/15.Zhou Yujiao Family Genetic Testing Report.pdf]

遗传性疾病基因检测补充报告（一代验证）

样本信息

样本编号	检测类型	送检单位	报告日期
20Y01164	再分析	桂林医学院附属医院	2020-07-02

验证者信息

姓名	与受检者关系	性别	年龄	样本类型	送检日期
周祥兴	父亲	男	57周岁	外周血	2020-06-27
周祥银	母亲	女	55周岁	外周血	2020-06-27
王支桂	弟弟	男	33周岁	外周血	2020-06-27

验证位点结果

基因	突变位置	基因亚区	HGVS	突变类型	验证结果
TRPV4	chr12:109794328-109794328	exon8	NM_021625.4: c.1491+1G>A: .	splicing	父亲：杂合<br>母亲：野生型<br>弟弟：野生型

· 表示数据库无收录。参考数据库版本为：Human Genome 38 (hg38/GRCh38)。

SNV 及 InDel 结果图

TRPV4:NM_021625.4:exon8:c.1491+1G>A:.

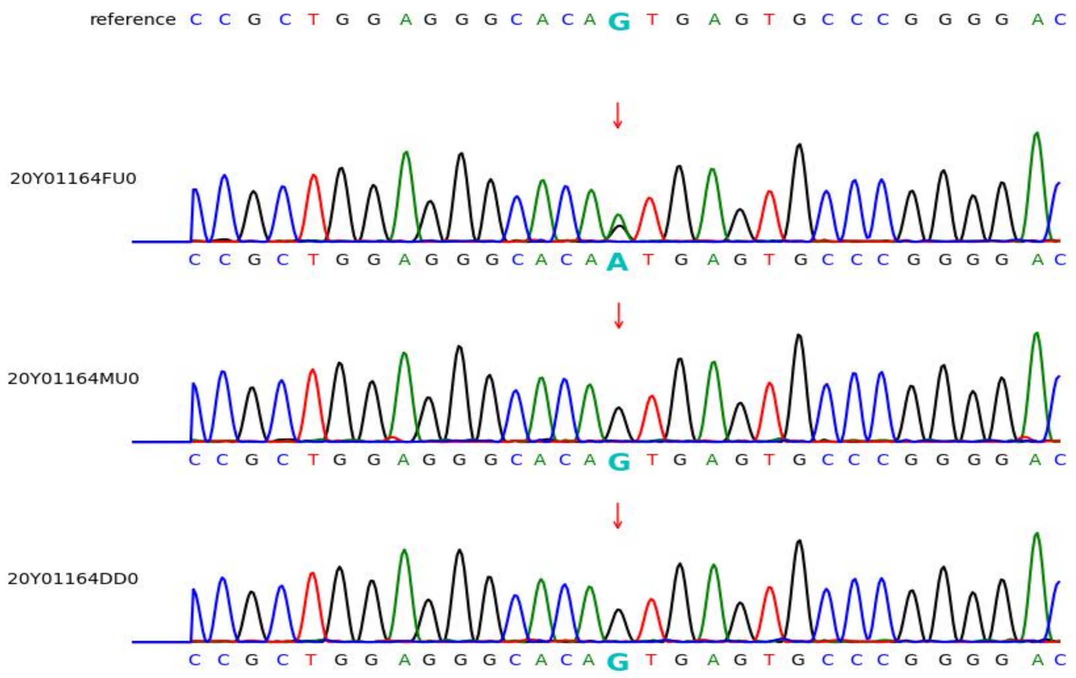

图注: 20Y01164FU0:父亲; 20Y01164MU0:母亲; 20Y01164DD0:弟弟
