# Supplementary material for: Potential role of the Trpv4 c.1491+1G>A mutation in pulmonary fibrosis in a gene-edited mouse model
Source: Front Genet. 2026 Jun 18;17:1834091. doi: 10.3389/fgene.2026.1834091 (PMC13322678; doi:10.3389/fgene.2026.1834091)
Supplement: Supplementary file 4 [file DataSheet2.zip › Supplementary.2/5.Table S3.docx]

**Supplementary Table S3**

**Trpv4 CDS Sequence-Editing:**ATGGCAGATCCTGGTGATGGTCCCCGTGCAGCGCCTGGGGAGGTGGCTGAGCCCCCTGGAGATGAGAGTGGTACCTCTGGTGGGGAGGCCTTCCCCCTCTCTTCCCTGGCCAATCTGTTTGAGGGGGAGGAAGGCTCCTCTTCTCTTTCCCCGGTGGATGCTAGCCGCCCTGCTGGCCCTGGCGATGGACGTCCAAACCTGCGTATGAAGTTCCAGGGCGCTTTCCGCAAGGGGGTTCCCAACCCCATTGACCTGTTGGAGTCCACCCTGTACGAGTCCTCAGTAGTGCCTGGGCCCAAGAAAGCGCCCATGGATTCCTTGTTCGACTACGGCACTTACCGTCACCACCCCAGTGACAACAAGAGATGGAGGAGAAAGGTCGTGGAGAAGCAGCCACAGAGCCCCAAAGCTCCTGCACCCCAGCCACCCCCCATCCTCAAAGTCTTCAATCGGCCCATCCTCTTTGACATTGTGTCCCGGGGCTCCACTGCGGACCTAGATGGACTGCTCTCCTTCTTGTTGACCCACAAGAAGCGCCTGACTGATGAGGAGTTCCGGGAGCCGTCCACGGGGAAGACCTGCCTGCCCAAGGCGCTGCTGAACCTAAGCAACGGGCGCAACGACACCATCCCGGTGTTGCTGGACATTGCGGAGCGCACCGGCAACATGCGTGAATTCATCAACTCGCCCTTCAGAGACATCTACTACCGAGGCCAGACATCCCTGCACATTGCCATCGAACGGCGCTGCAAGCACTACGTGGAGCTGCTGGTGGCCCAGGGAGCCGACGTGCACGCCCAGGCCCGCGGCCGCTTCTTCCAGCCCAAGGATGAGGGAGGCTACTTCTACTTTGGGGAGCTGCCCTTGTCCCTGGCAGCCTGCACCAACCAGCCGCACATCGTCAACTACCTGACAGAGAACCCTCACAAGAAAGCTGACATGAGGCGACAGGACTCGAGGGGGAACACGGTGCTGCACGCGCTGGTGGCCATCGCCGACAACACCCGAGAGAACACCAAGTTTGTCACCAAGATGTACGACCTGCTGCTTCTCAAGTGTTCACGCCTCTTCCCCGACAGCAACCTGGAGACAGTTCTCAACAATGATGGCCTTTCGCCTCTCATGATGGCTGCCAAGACAGGCAAGATCGGGGTCTTTCAGCACATCATCCGACGTGAGGTGACAGATGAGGACACCCGGCATCTGTCTCGCAAGTTCAAGGACTGGGCCTATGGGCCTGTGTATTCTTCTCTCTACGACCTCTCCTCCCTGGACACATGCGGGGAGGAGGTGTCCGTGCTGGAGATCCTGGTGTACAACAGCAAGATCGAGAACCGCCATGAGATGCTGGCTGTAGAGCCCATTAACGAACTGTTGAGAGACAAGTGGCGTAAGTTTGGGGCTGTGTCCTTCTACATCAACGTGGTCTCCTATCTGTGTGCCATGGTCATCTTCACCCTCACCGCCTACTATCAGCCACTGGAGGGCACGATGAGTGCTCACTGGGGAGCCGGCACAAGGACAGCCTCTGCAGCGGGACAGAAGACAGCTTGGGGGTGGGGAGGAAGATGCCCAGCACCCAGATGGCTCAGCCTGGAGAGACTTTGGGGGAGACAGGAGAGGGCGCTGCTGAGGACGCAGATGTTTGGGGCAGCAGGGAGCTGGTGTTGGGGGCAGGGGAGGACCTGTTGCATCTGCTCTTCCCGCCCTGCCCCCTCAGCCACCCTACCCTTACCGGACCACAGTGGACTACCTGAGGCTGGCTGGCGAGGTCATCACGCTCTTCACAGGAGTCCTGTTCTTCTTTACCAGTATCAAAGACTTGTTCACGAAGAAATGCCCTGGAGTGAATTCTCTCTTCGTCGATGGCTCCTTCCAGTTACTCTACTTCATCTACTCTGTGCTGGTGGTTGTCTCTGCGGCGCTCTACCTGGCTGGGATCGAGGCCTACCTGGCTGTGATGGTCTTTGCCCTGGTCCTGGGCTGGATGAATGCGCTGTACTTCACGCGCGGGTTGAAGCTGACGGGGACCTACAGCATCATGATTCAGAAGATCCTCTTCAAAGACCTCTTCCGCTTCCTGCTTGTGTACCTGCTCTTCATGATCGGCTATGCCTCAGCCCTGGTCACCCTCCTGAATCCGTGCACCAACATGAAGGTCTGTGA

**Trpv4 CDS Sequence-WT:**ATGGCAGATCCTGGTGATGGTCCCCGTGCAGCGCCTGGGGAGGTGGCTGAGCCCCCTGGAGATGAGAGTGGTACCTCTGGTGGGGAGGCCTTCCCCCTCTCTTCCCTGGCCAATCTGTTTGAGGGGGAGGAAGGCTCCTCTTCTCTTTCCCCGGTGGATGCTAGCCGCCCTGCTGGCCCTGGCGATGGACGTCCAAACCTGCGTATGAAGTTCCAGGGCGCTTTCCGCAAGGGGGTTCCCAACCCCATTGACCTGTTGGAGTCCACCCTGTACGAGTCCTCAGTAGTGCCTGGGCCCAAGAAAGCGCCCATGGATTCCTTGTTCGACTACGGCACTTACCGTCACCACCCCAGTGACAACAAGAGATGGAGGAGAAAGGTCGTGGAGAAGCAGCCACAGAGCCCCAAAGCTCCTGCACCCCAGCCACCCCCCATCCTCAAAGTCTTCAATCGGCCCATCCTCTTTGACATTGTGTCCCGGGGCTCCACTGCGGACCTAGATGGACTGCTCTCCTTCTTGTTGACCCACAAGAAGCGCCTGACTGATGAGGAGTTCCGGGAGCCGTCCACGGGGAAGACCTGCCTGCCCAAGGCGCTGCTGAACCTAAGCAACGGGCGCAACGACACCATCCCGGTGTTGCTGGACATTGCGGAGCGCACCGGCAACATGCGTGAATTCATCAACTCGCCCTTCAGAGACATCTACTACCGAGGCCAGACATCCCTGCACATTGCCATCGAACGGCGCTGCAAGCACTACGTGGAGCTGCTGGTGGCCCAGGGAGCCGACGTGCACGCCCAGGCCCGCGGCCGCTTCTTCCAGCCCAAGGATGAGGGAGGCTACTTCTACTTTGGGGAGCTGCCCTTGTCCCTGGCAGCCTGCACCAACCAGCCGCACATCGTCAACTACCTGACAGAGAACCCTCACAAGAAAGCTGACATGAGGCGACAGGACTCGAGGGGGAACACGGTGCTGCACGCGCTGGTGGCCATCGCCGACAACACCCGAGAGAACACCAAGTTTGTCACCAAGATGTACGACCTGCTGCTTCTCAAGTGTTCACGCCTCTTCCCCGACAGCAACCTGGAGACAGTTCTCAACAATGATGGCCTTTCGCCTCTCATGATGGCTGCCAAGACAGGCAAGATCGGGGTCTTTCAGCACATCATCCGACGTGAGGTGACAGATGAGGACACCCGGCATCTGTCTCGCAAGTTCAAGGACTGGGCCTATGGGCCTGTGTATTCTTCTCTCTACGACCTCTCCTCCCTGGACACATGCGGGGAGGAGGTGTCCGTGCTGGAGATCCTGGTGTACAACAGCAAGATCGAGAACCGCCATGAGATGCTGGCTGTAGAGCCCATTAACGAACTGTTGAGAGACAAGTGGCGTAAGTTTGGGGCTGTGTCCTTCTACATCAACGTGGTCTCCTATCTGTGTGCCATGGTCATCTTCACCCTCACCGCCTACTATCAGCCACTGGAGGGCACGCCACCCTACCCTTACCGGACCACAGTGGACTACCTGAGGCTGGCTGGCGAGGTCATCACGCTCTTCACAGGAGTCCTGTTCTTCTTTACCAGTATCAAAGACTTGTTCACGAAGAAATGCCCTGGAGTGAATTCTCTCTTCGTCGATGGCTCCTTCCAGTTACTCTACTTCATCTACTCTGTGCTGGTGGTTGTCTCTGCGGCGCTCTACCTGGCTGGGATCGAGGCCTACCTGGCTGTGATGGTCTTTGCCCTGGTCCTGGGCTGGATGAATGCGCTGTACTTCACGCGCGGGTTGAAGCTGACGGGGACCTACAGCATCATGATTCAGAAGATCCTCTTCAAAGACCTCTTCCGCTTCCTGCTTGTGTACCTGCTCTTCATGATCGGCTATGCCTCAGCCCTGGTCACCCTCCTGAATCCGTGCACCAACATGAAGGTCTGTGACGAGGACCAGAGCAACTGCACGGTGCCCACGTATCCTGCGTGCCGCGACAGCGAGACCTTCAGCGCCTTCCTCCTGGACCTCTTCAAGCTCACCATCGGCATGGGAGACCTGGAGATGCTGAGCAGCGCCAAGTACCCCGTGGTCTTCATCCTCCTGCTGGTCACCTACATCATCCTCACCTTCGTGCTCCTGTTGAACATGCTTATCGCCCTCATGGGTGAGACCGTGGGCCAGGTGTCCAAGGAGAGCAAGCACATCTGGAAGTTGCAGTGGGCCACCACCATCCTGGACATCGAGCGTTCCTTCCCTGTGTTCCTGAGGAAGGCCTTCCGCTCCGGAGAGATGGTGACTGTGGGCAAGAGCTCAGATGGCACTCCGGACCGCAGGTGGTGCTTCAGGGTGGACGAGGTGAACTGGTCTCACTGGAACCAGAACTTGGGCATCATTAACGAGGACCCTGGCAAGAGTGAAATCTACCAGTACTATGGCTTCTCCCACACCGTGGGGCGCCTTCGTAGGGATCGTTGGTCCTCGGTGGTGCCCCGCGTAGTGGAGCTGAACAAGAACTCAAGCGCAGATGAAGTGGTGGTACCCCTGGATAACCTAGGGAACCCCAACTGTGACGGCCACCAGCAGGGCTACGCTCCCAAGTGGAGGACGGACGATGCCCCACTGTAG

**TRPV4 Protein Sequence-Editing:**MADPGDGPRAAPGEVAEPPGDESGTSGGEAFPLSSLANLFEGEEGSSSLSPVDASRPAGPGDGRPNLRMKFQGAFRKGVPNPIDLLESTLYESSVVPGPKKAPMDSLFDYGTYRHHPSDNKRWRRKVVEKQPQSPKAPAPQPPPILKVFNRPILFDIVSRGSTADLDGLLSFLLTHKKRLTDEEFREPSTGKTCLPKALLNLSNGRNDTIPVLLDIAERTGNMREFINSPFRDIYYRGQTSLHIAIERRCKHYVELLVAQGADVHAQARGRFFQPKDEGGYFYFGELPLSLAACTNQPHIVNYLTENPHKKADMRRQDSRGNTVLHALVAIADNTRENTKFVTKMYDLLLLKCSRLFPDSNLETVLNNDGLSPLMMAAKTGKIGVFQHIIRREVTDEDTRHLSRKFKDWAYGPVYSSLYDLSSLDTCGEEVSVLEILVYNSKIENRHEMLAVEPINELLRDKWRKFGAVSFYINVVSYLCAMVIFTLTAYYQPLEGTMSAHWGAGTRTASAAGQKTAWGWGGRCPAPRWLSLERLWGRQERALLRTQMFGAAGSWCWGQGRTCCICSSRPAPSATLPLPDHSGLPEAGWRGHHALHRSPVLLYQYQRLVHEEMPWSEFSLRRWLLPVTLLHLLCAGGCLCGALPGWDRGLPGCDGLCPGPGLDECAVLHARVEADGDLQHHDSEDPLQRPLPLPACVPALHDRLCLSPGHPPESVHQHEGL

**TRPV4 Protein Sequence-WT:**MADPGDGPRAAPGEVAEPPGDESGTSGGEAFPLSSLANLFEGEEGSSSLSPVDASRPAGPGDGRPNLRMKFQGAFRKGVPNPIDLLESTLYESSVVPGPKKAPMDSLFDYGTYRHHPSDNKRWRRKVVEKQPQSPKAPAPQPPPILKVFNRPILFDIVSRGSTADLDGLLSFLLTHKKRLTDEEFREPSTGKTCLPKALLNLSNGRNDTIPVLLDIAERTGNMREFINSPFRDIYYRGQTSLHIAIERRCKHYVELLVAQGADVHAQARGRFFQPKDEGGYFYFGELPLSLAACTNQPHIVNYLTENPHKKADMRRQDSRGNTVLHALVAIADNTRENTKFVTKMYDLLLLKCSRLFPDSNLETVLNNDGLSPLMMAAKTGKIGVFQHIIRREVTDEDTRHLSRKFKDWAYGPVYSSLYDLSSLDTCGEEVSVLEILVYNSKIENRHEMLAVEPINELLRDKWRKFGAVSFYINVVSYLCAMVIFTLTAYYQPLEGTPPYPYRTTVDYLRLAGEVITLFTGVLFFFTSIKDLFTKKCPGVNSLFVDGSFQLLYFIYSVLVVVSAALYLAGIEAYLAVMVFALVLGWMNALYFTRGLKLTGTYSIMIQKILFKDLFRFLLVYLLFMIGYASALVTLLNPCTNMKVCDEDQSNCTVPTYPACRDSETFSAFLLDLFKLTIGMGDLEMLSSAKYPVVFILLLVTYIILTFVLLLNMLIALMGETVGQVSKESKHIWKLQWATTILDIERSFPVFLRKAFRSGEMVTVGKSSDGTPDRRWCFRVDEVNWSHWNQNLGIINEDPGKSEIYQYYGFSHTVGRLRRDRWSSVVPRVVELNKNSSADEVVVPLDNLGNPNCDGHQQGYAPKWRTDDAPL
